# Fatty acid oxidation of alternatively activated macrophages prevents foam cell formation, but *Mycobacterium tuberculosis* counteracts this process *via* HIF-1α activation

**DOI:** 10.1371/journal.ppat.1008929

**Published:** 2020-10-01

**Authors:** Melanie Genoula, José Luis Marín Franco, Mariano Maio, Belén Dolotowicz, Malena Ferreyra, M. Ayelén Milillo, Rémi Mascarau, Eduardo José Moraña, Domingo Palmero, Mario Matteo, Federico Fuentes, Beatriz López, Paula Barrionuevo, Olivier Neyrolles, Céline Cougoule, Geanncarlo Lugo-Villarino, Christel Vérollet, María del Carmen Sasiain, Luciana Balboa

**Affiliations:** 1 Instituto de Medicina Experimental (IMEX)-CONICET, Academia Nacional de Medicina, Buenos Aires, Argentina; 2 International Associated Laboratory (LIA) CNRS IM-TB/HIV (1167), Buenos Aires, Argentina—Toulouse, France; 3 Institut de Pharmacologie et de Biologie Structurale, Université de Toulouse, CNRS, UPS, Toulouse, France; 4 Instituto Prof. Dr. Raúl Vaccarezza, Hospital de Infecciosas Dr. F.J. Muñiz, Buenos Aires, Argentina; 5 Laboratorio de Tuberculosis y Micobacteriosis “Dr. Abel Cetrángolo”, Hospital de Infecciosas Dr. F.J. Muñiz, Buenos Aires, Argentina; 6 Instituto Nacional de Enfermedades Infecciosas (INEI), ANLIS "Carlos G. Malbrán, Buenos Aires, Argentina; New Jersey Medical School, UNITED STATES

## Abstract

The ability of *Mycobacterium tuberculosis* (Mtb) to persist inside host cells relies on metabolic adaptation, like the accumulation of lipid bodies (LBs) in the so-called foamy macrophages (FM), which are favorable to Mtb. The activation state of macrophages is tightly associated to different metabolic pathways, such as lipid metabolism, but whether differentiation towards FM differs between the macrophage activation profiles remains unclear. Here, we aimed to elucidate whether distinct macrophage activation states exposed to a tuberculosis-associated microenvironment or directly infected with Mtb can form FM. We showed that the triggering of signal transducer and activator of transcription 6 (STAT6) in interleukin (IL)-4-activated human macrophages (M(IL-4)) prevents FM formation induced by pleural effusion from patients with tuberculosis. In these cells, LBs are disrupted by lipolysis, and the released fatty acids enter the β-oxidation (FAO) pathway fueling the generation of ATP in mitochondria. Accordingly, murine alveolar macrophages, which exhibit a predominant FAO metabolism, are less prone to become FM than bone marrow derived-macrophages. Interestingly, direct infection of M(IL-4) macrophages with Mtb results in the establishment of aerobic glycolytic pathway and FM formation, which could be prevented by FAO activation or inhibition of the hypoxia-inducible factor 1-alpha (HIF-1α)-induced glycolytic pathway. In conclusion, our results demonstrate that Mtb has a remarkable capacity to induce FM formation through the rewiring of metabolic pathways in human macrophages, including the STAT6-driven alternatively activated program. This study provides key insights into macrophage metabolism and pathogen subversion strategies.

## Author summary

Tuberculosis is a deadly disease caused by *Mycobacterium tuberculosis*. Although progress has been made in tuberculosis control, there are unexplored aspects of how the immune system deals with the pathogen that need to be addressed. *M*. *tuberculosis* primarily lives in macrophages, immune cells which can destroy mycobacteria. In spite of the multiple microbicidal properties, the bacterium still manipulates the metabolism of macrophages, reflected in the accumulation lipid droplets and consequent differentiation into foamy macrophages. These lipid-laden macrophages constitute a favorable niche for the bacteria to persist hidden from our immune defense. Macrophages are classified into different programs depending on how they are activated by environmental signals, ranging from classically (microbicidal/inflammatory) to alternatively (tissue remodeling/repair/growth) activated spectrum extremes. We found that, unlike other programs, alternatively activated macrophages are reluctant to accumulate lipid droplets due to the signal transducer and activator of transcription 6 (STAT6), which promotes the degradation of those lipids. Notwithstanding, *M*. *tuberculosis* counteracts lipolysis by rewiring the metabolism of alternatively activated macrophages towards the accumulation of lipid droplets via the activation of the hypoxia-inducible factor 1-alpha (HIF-1α). Therefore, this study contributes to the better understanding of how bacillus shifts the metabolism of macrophages, which can be crucial to therapeutic purposes.

## Introduction

Tuberculosis (TB) is a highly contagious disease caused by *Mycobacterium tuberculosis* (Mtb). Even though the treatment of the disease is standardized, TB still remains one of the top 10 causes of death worldwide [1]. Chronic host-pathogen interaction in TB leads to extensive metabolic remodeling in both the host and the pathogen [2]. The success of Mtb as a pathogen is mainly due to its efficient adaptation to the intracellular milieu of human macrophages, leading to intrinsic metabolic changes in these cells. One of these changes is the dysregulation of the lipid metabolism, which induces the formation of foamy macrophages (FM). FM are cells filled with lipid bodies (LBs) that are abundant in granulomatous structures in both experimentally infected animals and patients [3,4], and fail to control the infection [5]. Recently, we used the pleural effusions (PE) from TB patients (TB-PE) as a tool to recapitulate human lung TB-associated microenvironment, and demonstrated that uninfected-macrophages exposed to TB-PE form FM displaying an immunosuppressive profile though the activation of the interleukin (IL)-10/STAT3 axis [6].

It is widely accepted that macrophages undergo different activation programs by which they carry out unique physiological and defensive functions. Essentially, macrophages can modify their metabolic functions from one end of the spectrum characterized by healing/repairing/growth functions (M2 macrophages) towards the other end exhibiting a killing/inhibitory/microbicidal functional profile (M1 macrophages) [7,8]. M1 macrophages, generally induced by interferon (IFN)-γ and/or lipopolysaccharide (LPS) stimulation, are endowed with microbicidal properties; M2 macrophages, usually differentiated upon IL-4 or IL-13 stimulation, are reported to be immunomodulatory and poorly microbicidal, resulting in impaired anti-mycobacterial properties [9–11]. In addition, while M1 macrophages rely on aerobic glycolysis dependent on the hypoxia-inducible factor 1-alpha (HIF-1α) activation [12], M2 macrophages require the induction of fatty acid oxidation (FAO), at least in the murine model [13–16], through signal transducer and activator of transcription 6 (STAT6) activation [17]. FAO is the mitochondrial process of breaking down a fatty acid into acetyl-CoA units requiring the carnitine palmitoyltransferase (CPT) system, which consists of a transporter and the CPT1/CPT2 mitochondrial membrane enzymes [18]. This facilitates the transport of long-chain fatty acids into the mitochondrial matrix, where they can be metabolized by the oxidative phosphorylation (OXPHOS) pathway and produce ATP. Moreover, in the context of the murine model of Mtb infection, macrophages were recently shown to be pre-programmed towards different metabolic pathways, according to their ontogeny. Indeed, while lung interstitial macrophages (adult hematopoietic origin) are glycolytically active and capable of controlling Mtb infection, resident alveolar macrophages (fetal liver origin) are committed to FAO and OXPHOS bearing higher bacillary loads [19].

Considering that mitochondria are the main site of lipid degradation, and that mitochondrial metabolic functions are known to differ between macrophage profiles, we investigated if the different activation programs of human macrophages differ in their ability to form FM in the context of TB. To this end, we employed our already characterized TB-PE model to induce FM differentiation [6], and explored the molecular and metabolic pathways capable of altering this process. Indeed, the PE is an excess of fluid recovered from pleural space characterized by a high protein content and specific leukocytes [20]. We have previously demonstrated that the TB-PE tilted *ex vivo* human monocyte differentiation towards an anti-inflammatory M2-like macrophage activation, reproducing the phenotype exhibited by macrophages directly isolated from the pleural cavity of TB patients and from lung biopsies of non-human primates with advanced TB [21]. Likewise, we showed that the acellular fraction of TB-PE modified the lipid metabolism of human macrophages, resulting in FM formation and diminished effector functions against the bacilli [6]. In this study, we demonstrated that IL-4/STAT6-driven FAO prevents FM differentiation when exposed to TB-PE and, conversely, we revealed that Mtb infection counteracts it by inhibiting HIF-1α to establish the formation of FM. Therefore, our study contributes to a better understanding of how alterations of the host metabolic pathways affect pathogen persistence.

## Results

### STAT6 activation in M(IL-4) macrophages prevents the formation of foamy macrophages induced by a TB-associated microenvironment

**Fig 1:**
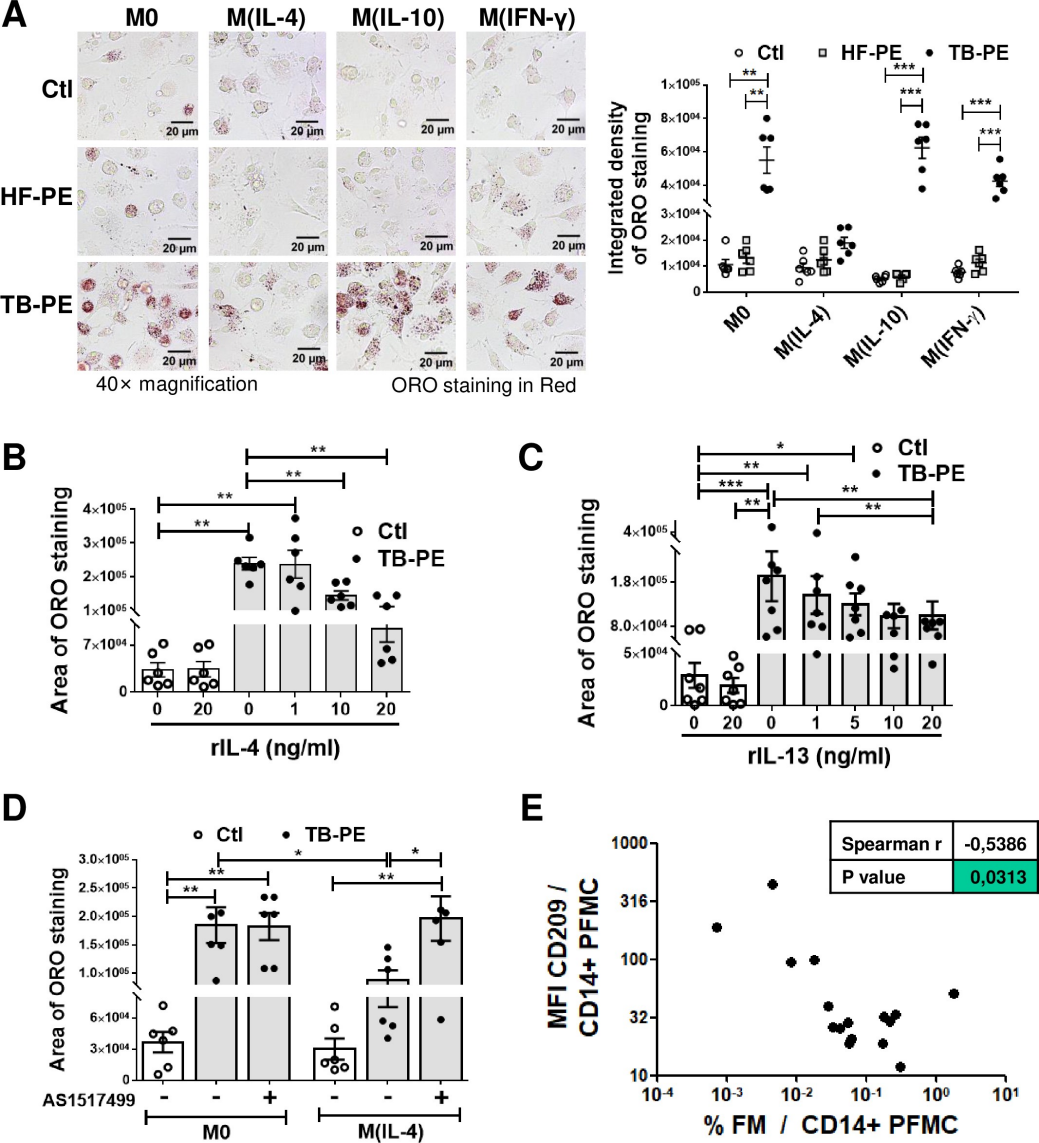
The IL-4/STAT6 Axis prevents the formation of foamy macrophages. Human macrophages were left untreated (M0) or polarized with either IL-4 (M(IL-4)), IL-10 (M(IL-10)) or IFN-γ (M(IFN-γ)) for 48 h, treated or not with the acellular fraction of TB pleural effusions (TB-PE) or heart-failure-associated effusions (HF-PE) for 24 h and then stained with Oil Red O (ORO). **(A)** Left panel: Representative images (40× magnification), right panel: the integrated density of ORO staining. **(B-C)** Quantification of area of ORO staining of macrophages polarized with either different doses of recombinant IL-4 (B) or IL-13 (C) for 48 h and exposed to TB-PE for further 24 h. **(D)** Quantification of area of ORO staining of M0 and M(IL-4) macrophages treated with TB-PE and exposed or not to AS1517499, a chemical inhibitor of STAT6. Values are expressed as means ± SEM of six independent experiments, considering five microphotographs per experiment. **(E)** Correlation study between the mean fluorescence intensity (MFI) of CD209 cell-surface expression in CD14^+^ cells from TB pleural cavity and the percentage of lipid-laden CD14^+^ cells within the pleural fluids mononuclear cells (PFMC) (n = 16) found in individual preparations of TB-PE. Spearman’s rank test. Friedman test followed by Dunn’s Multiple Comparison Test: *p<0.05; **p<0.01; ***p<0.001 as depicted by lines.

In order to evaluate whether different activation programs in human macrophages differ in their ability to form FM upon treatment with the acellular fraction of TB-PE, macrophages were differentiated with IL-4, IL-10 or IFN-γ, and treated (or not) with TB-PE. The intracellular accumulation of LBs was then determined by Oil Red O (ORO) staining. The profiles of macrophage activation were evaluated by determining the expression of cell-surface and molecular markers (**S1A and S1B Fig**). As expected, TB-PE induced FM formation in non-polarized (M0) macrophages (**Fig 1A**) [6]. Interestingly, TB-PE also induced the accumulation of LBs in M(IFN-γ) and M(IL-10), but not in M(IL-4) macrophages, suggesting that the macrophage activation program influences LBs formation by TB-PE. In agreement with our previous work [6], the accumulation of LBs observed in M0, M(IFN-γ) and M(IL-10) cells was specific for TB-PE treatment given that PE from patients with heart-failure (HF-PE) failed to do so (**Fig 1A**). Moreover, the addition of cytokines known to drive the alternative activation program in macrophages, *i*.*e*. recombinant IL-4 or IL-13 (**S2A Fig**), inhibited LBs accumulation in TB-PE-treated M0 in a dose-dependent manner (**Fig 1B and 1C and S2B and S2C Fig**). Since IL-4/IL-13-dependent triggering of STAT6 is required for alternatively activated macrophages [22,23], we evaluated the impact of the chemical AS1517499 agent, which prevents STAT6 phosphorylation (**S2D and S2E Fig** and [24]), in LBs accumulation. Inhibition of STAT6 activation enabled M(IL-4) cells to accumulate LBs (**Fig 1D**).

In order to evaluate whether M(IL-4) macrophages are less prone to become foamy *in vivo* during TB, we assessed the phenotype of CD14^+^ cells and incidence of FM found within the pleural cavity of TB patients. We noticed that the expression levels of an IL-4-driven marker signature, such as CD209/DC-SIGN, was negatively correlated with the percentage of FM found among the pleural fluid mononuclear cells, unlike other cell-surface receptors (**Fig 1E and S3 Fig**). Noticeably, we observed a positive correlation between the percentage of FM and the expression of MerTK, a M(IL-10)-associated marker (**S3A Fig**), which confirms our previous results showing that IL-10 enhances FM formation in TB-PE-treated macrophages [6]. These results indicate that the IL-4-driven phenotype prevents the foamy program in human macrophages in the context of a natural TB-associated microenvironment. Therefore, the IL-4/STAT6 signaling pathway mediates the inhibition of FM formation induced by TB-PE.

### The biogenesis of LBs is not impaired in TB-PE-treated M(IL-4) macrophages

**Fig 2:**
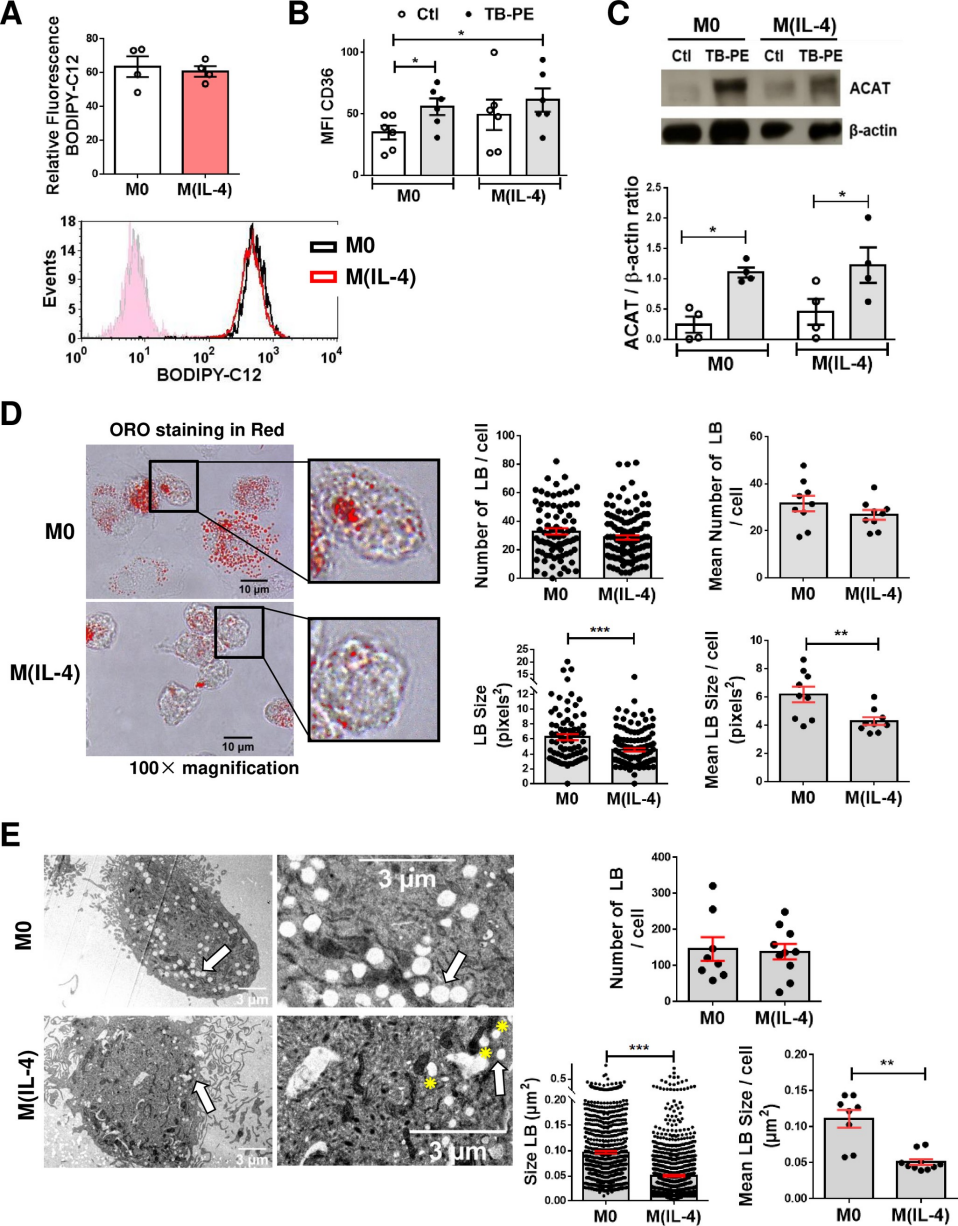
The biogenesis of LBs is not impaired in TB-PE-treated M(IL-4) macrophages. **(A)** Human macrophages were left untreated (M0) or polarized with IL-4 (M(IL-4)) for 48 h and then exposed or not to the red-labelled saturated fatty acid C12 (BODIPY C12) for 1 min. Upper panel: relative BODIPY C12 fluorescence, lower panel: representative histogram. Values are expressed as means ± SEM of four independent experiments. **(B)** Median fluorescence intensity (MFI) of CD36 measured by flow cytometry in M0 and M(IL-4) macrophages exposed or not to TB-PE. Values are expressed as means ± SEM of six independent experiments. Friedman test followed by Dunn’s Multiple Comparison Test: *p<0.05 for experimental condition vs Ctl. **(C)** Upper panel: analysis of ACAT and β-actin protein level by Western Blot; lower panel: quantification in M0 and M(IL-4) macrophages treated or not with TB-PE for 24 h (n = 4). Wilcoxon signed rank test: *p<0.05 as depicted by lines. **(D)** Morphometric analysis of LBs in ORO-labelled TB-PE-treated M0 and M(IL-4) macrophages. Representative images of ORO staining (100× magnification) are shown. Number of LBs (upper left panel) and size (lower left panel) per cell are shown, and their mean is represented by red bars. Mann Whitney test: ***p<0.001. Mean number of LBs (upper right panel) and mean size (lower right panel) per cell per donor (n = 9 donors). Each determination represents the mean of 20–40 individual cells per donor. Wilcoxon signed rank test: **p<0.01. **(E)** Electron microscopy micrographs of TB-PE-treated M0 and M(IL-4) macrophages showing LBs (white arrows) and LBs nearby mitochondria (yellow asterisks). Upper panel: numbers of LBs per cell; Lower panels: size of LBs in TB-PE-treated M0 and M(IL-4) macrophages considering all LBs`size determinations (left) or the mean of 8–10 individual cells of one representative donor (right). Mann Whitney test: **p<0.01; ***p<0.001.

In order to elucidate how the IL-4/STAT6 axis could interfere with TB-PE-induced macrophage differentiation into FM, we investigated cellular mechanisms associated with LBs biogenesis. We found that M0 and M(IL-4) macrophages did not differ in their ability to uptake fatty acids (**Fig 2A**). In addition, TB-PE was able to induce the expression of the scavenger receptor CD36, which binds long-chain fatty acids and facilitates their transport into cells (**Fig 2B**), and that of Acyl-CoA cholesterol acyltransferase (ACAT), the enzyme that esterifies free cholesterol (**Fig 2C**). In line with our previous study showing that TB-PE drives a STAT3-dependent induction of ACAT expression in M0 [6], herein we observed that the phosphorylated form of STAT3 is increased in both M0 and M(IL-4) macrophages in response to TB-PE (**S3B Fig**). We next evaluated the morphometric features of the LBs formed in TB-PE-stimulated M0 and M(IL-4) cells. As shown in **Fig 2D**, the analysis of ORO-stained images suggests that, despite the fact that LBs were formed in M(IL-4) cells, they were significantly smaller. As the analysis of ORO-stained images are not optimal to enumerate LBs exhaustively, we analysed the morphometric features by transmission electron microscopy and confirmed the presence of smaller LBs in TB-PE-treated M(IL-4) macrophages compared to M0 cells (**Fig 2E**, arrows). These results suggest that, while the biogenesis of LBs is not impaired in M(IL-4) macrophages, these cells are still reluctant to acquire a proper foamy phenotype that is typical of TB-PE treatment.

### Inhibition of lipolysis in M(IL-4) macrophages restores FM phenotype and impairs the control of Mtb intracellular growth

**Fig 3:**
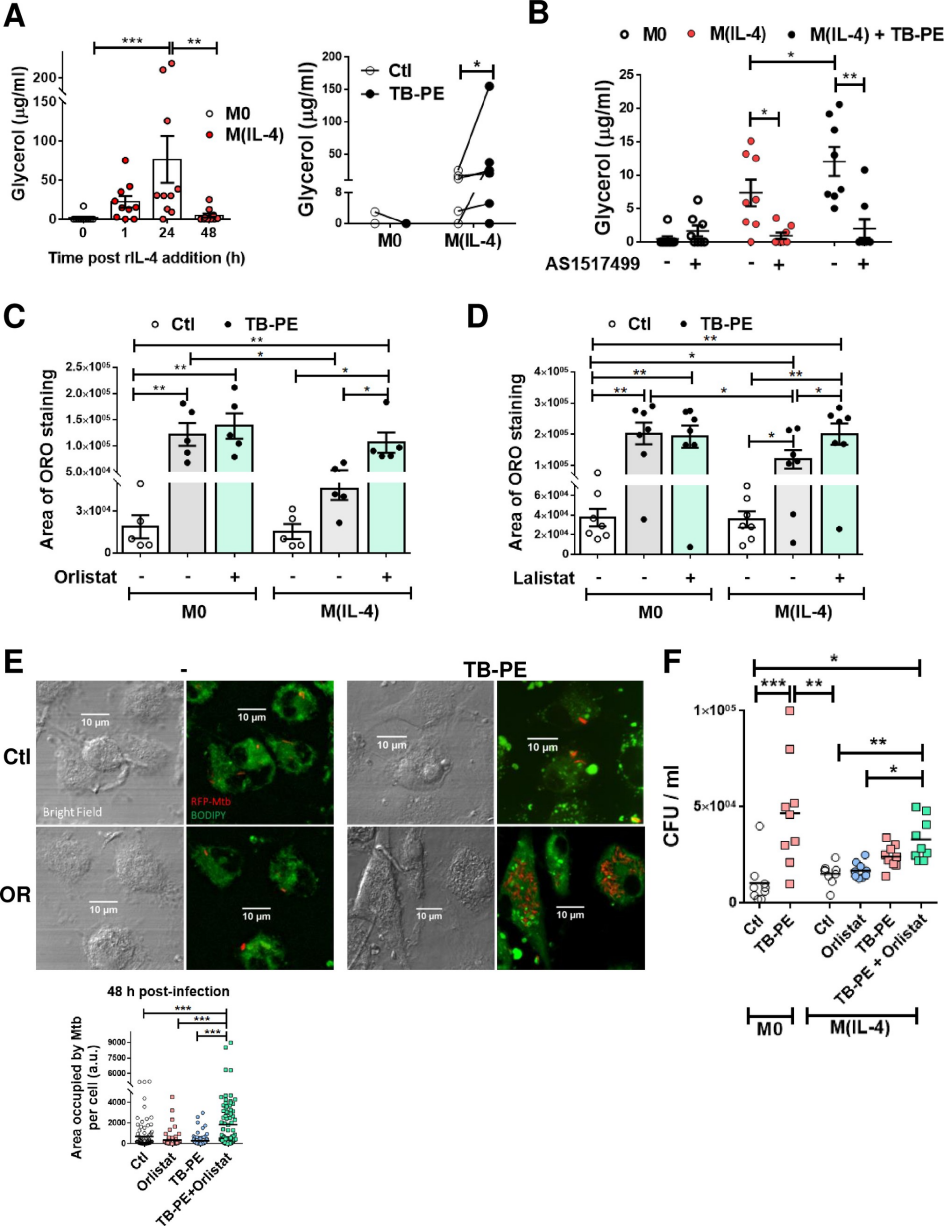
Enhanced lipolysis inhibits lipid bodies`accumulation in M(IL-4) macrophages. **(A)** Macrophages were polarized or not with IL-4 for 1, 24 or 48 h (left panel), and then treated or not with TB-PE for further 24 h (right panel). Thereafter, culture-medium was replaced by PBS containing 2% BSA (fatty acid-free), and supernatants were collected 6h later to measure the release of glycerol during this specific frame of time by colorimetric assays. Left panel: glycerol release during 6 h after 1, 24 or 48 h- cultures post-IL-4 addition (n = 10). Right panel: glycerol release after TB-PE treatment in M0 or M(IL-4) macrophages (n = 6). **(B)** Glycerol release by macrophages exposed or not to AS1517499 (n = 8). Values are expressed as means ± SEM of independent experiments. **(C-D)** ORO staining of M0 and M(IL-4) macrophages treated with TB-PE and exposed or not to either Orlistat (C) or Lalistat (D). Values are expressed as means ± SEM of five (C) or seven (D) independent experiments, considering five microphotographs per experiment. **(E-F)** M(IL-4) macrophages were treated or not with TB-PE in the presence or not of Orlistat for 24 h, washed and infected with RFP-Mtb (MOI of 5 bacteria/cell) **(E)** or unlabeled Mtb (MOI of 0.2 bacteria/cell) **(F)**. **(E)** Area with RFP-Mtb per cell in z-stacks from confocal laser scanning microscopy images at 48 h post-infection. Representative images are shown (60× magnification). Each determination represents individual cells of one donor. One way-ANOVA followed by Bonferroni test: ***p<0.001. **(F)** Intracellular colony forming units determined at 48 h post-infection (n = 9). Friedman followed by Dunn’s Multiple Comparison Test: *p<0.05; **p<0.01; ***p<0.001 as depicted by lines as depicted by lines.

As lysosomal lipolysis is essential for alternatively activated macrophages [14], we wondered whether lipolysis could be a mechanism by which IL-4/IL-13 reduce LBs accumulation induced by TB-PE. We found that both M(IL-4) and M(IL-13) cells displayed higher lipolytic activity than M0 cells, bearing the peak of lipolytic activity at 24 h after cytokine treatment, as measured by glycerol release (**Fig 3A left panel and S4A Fig**). Particularly, TB-PE-treated M(IL-4) cells released higher levels of glycerol than untreated M(IL-4) cells or TB-PE-treated M0 macrophages, indicating that more triglycerides were broken down in M(IL-4) macrophages upon TB-PE treatment **(Fig 3A right panel)**. Pharmacological inhibition of STAT6 activity reduced the release of glycerol in TB-PE-treated M(IL-4) cells (**Fig 3B**). Thereafter, we found that inhibition of lipase activity by the drugs Orlistat and Lalistat, which target neutral and acid lipolysis, respectively, enabled M(IL-4) macrophages to accumulate LBs and reduce glycerol release (**Fig 3C and 3D and S4B and S4C Fig**). Therefore, the activation of the IL4/STAT6 axis in M(IL-4) macrophages promotes lipolysis impairing the LBs accumulation by TB-PE treatment.

Next, we investigated whether the differentiation of M(IL-4) macrophages into FM could impact the control of Mtb intracellular growth. To this end, we determined the bacterial content in TB-PE-treated M(IL-4) macrophages, which were exposed (or not) to Orlistat prior to Mtb infection, based on two experimental approaches: i) assessing the area of red fluorescent protein (RFP)-Mtb associated to individual cells by confocal microscopy, and ii) enumerating the CFU recovered from the cultures. We confirmed that Orlistat treatment allows for the formation of FM in M(IL-4) cells exposed to TB-PE (**S4D Fig**) without decreasing the viability of Mtb-infected macrophages (**S4F Fig**). Importantly, while the entrance of mycobacteria at early time points was comparable among all conditions (**S4D and S4E Fig**), treatment of M(IL-4) cells with TB-PE in the presence of Orlistat rendered the cells more susceptible to Mtb intracellular replication (**Fig 3E and S4E Fig**). In the case of CFU counts, we observed a tendency for higher bacillary loads in TB-PE+Orlistat *versus* TB-PE-treated M(IL-4) macrophages, even though there is no statistical significance. However, a significant effect of Orlistat in the impairment of the control of Mtb infection was observed in the presence of TB-PE as a source of lipids (**Fig 3F**). Additionally, we confirmed our previous findings that the treatment with TB-PE in M0 macrophages, which become foamy, impaired the control of the bacillary load ([6] and **Fig 3F**). Therefore, inhibition of lipolysis prior to infection leads M(IL-4) cells to become foamy, and is associated with an increase in Mtb intracellular growth.

### The inhibition of fatty acid transport into mitochondria allows FM formation in TB-PE-treated M(IL-4) macrophages

**Fig 4:**
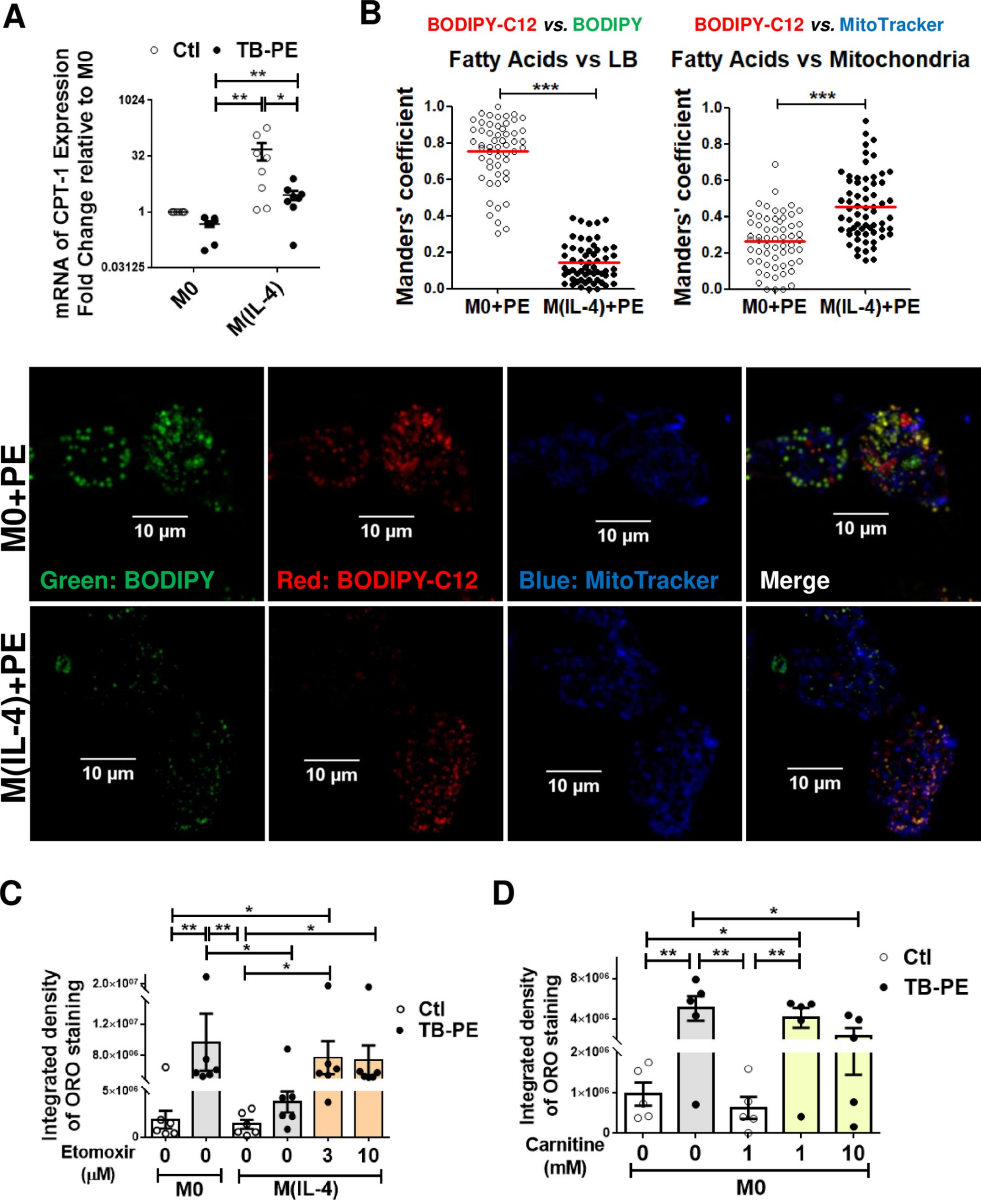
Inhibition of fatty acid transport into mitochondria allows lipids accumulation within LBs in TB-PE-treated M(IL-4) macrophages. M0 and M(IL-4) macrophages treated or not with TB-PE for 24 h **(A)** mRNA expression of *CPT1A* relative to M0 (n = 8). **(B)** Cells were pulsed with BODIPY Red C12 overnight during TB-PE treatment, then LBs were labeled using Green-BODIPY 493/503, and mitochondria were labeled using Far Red-MitoTracker. Quantification of the co-occurrence fractions of fatty acids (C12) with either LBs or Mitochondria in TB-PE-treated M0 and M(IL-4) macrophages were quantified by Manders' coefficient analysis. Mann Whitney test: ***p<0.001. Representative images of single and merged channels are shown. **(C)** ORO staining of M0 and M(IL-4) macrophages treated with TB-PE and exposed or not to Etomoxir. Values are expressed as means ± SEM of six independent experiments, considering five microphotographs per experiment. **(D)** ORO staining of M0 macrophages treated with TB-PE and exposed or not to L-carnitine. Values are expressed as means ± SEM of five independent experiments, considering five microphotographs per experiment. Friedman test followed by Dunn’s Multiple Comparison Test: *p<0.05; **p<0.01 for experimental condition vs Ctl or as depicted by lines.

Aside from storage in lipid droplets, fatty acids can also be transported into the mitochondria and be oxidized into acetyl-CoA by FAO. In this regard, the ultrastructural analysis of the cells revealed that LBs were frequently found nearby mitochondria in TB-PE-treated M(IL-4) macrophages (**Fig 2E**, asterisks). Therefore, we hypothesized that the enhanced lipolytic activity in M(IL-4) cells might drive fatty acids into the mitochondria destined for FAO, leading to high ATP production through the OXPHOS pathway. The expression of CPT1, an enzyme which is considered to be rate limiting for β-oxidation of long-chain fatty acids [18], was evaluated. As expected, we observed higher levels of *CPT1A* mRNA in M(IL-4) compared to M0 macrophages. Although the presence of TB-PE diminishes *CPT1A* expression, TB-PE-treated M(IL-4) cells still conserved higher levels than TB-PE-treated M0 cells (**Fig 4A**). Moreover, we performed a pulse-chase assay to track fluorescent fatty acids in relation to LBs and mitochondria in TB-PE-treated M0 and M(IL-4) macrophages. We found that, while the saturated fatty acid Red C12 was incorporated more often in neutral lipids within LBs in TB-PE-treated M0 cells, it was accumulated instead into mitochondria in TB-PE-treated M(IL-4) macrophages (**Fig 4B**).

We next decided to pharmacologically inhibit the activity of CPT1 and evaluate its impact on FM formation. We used an inhibitor of FAO (Etomoxir) in M(IL-4) cells at doses (3–10 μM) that do not provoke cell death (**S5A Fig**) or off-target effects [25]. CPT1 inhibition resulted in LBs accumulation in TB-PE-treated M(IL-4) cells **(Fig 4C**). In line with this, when we favored the FAO pathway by adding exogenous L-carnitine, which conjugates to fatty acids and allows them to enter the mitochondria [26], FM formation was inhibited in TB-PE-treated M0 cells **(Fig 4D and S5B Fig**). Altogether, these results indicate that IL-4 enhances fatty acid transport into the mitochondria, thus reducing lipid accumulation within LBs structures in TB-PE-treated M(IL-4) macrophages.

### Higher fatty acid metabolism in macrophages is associated with less LBs accumulation

**Fig 5:**
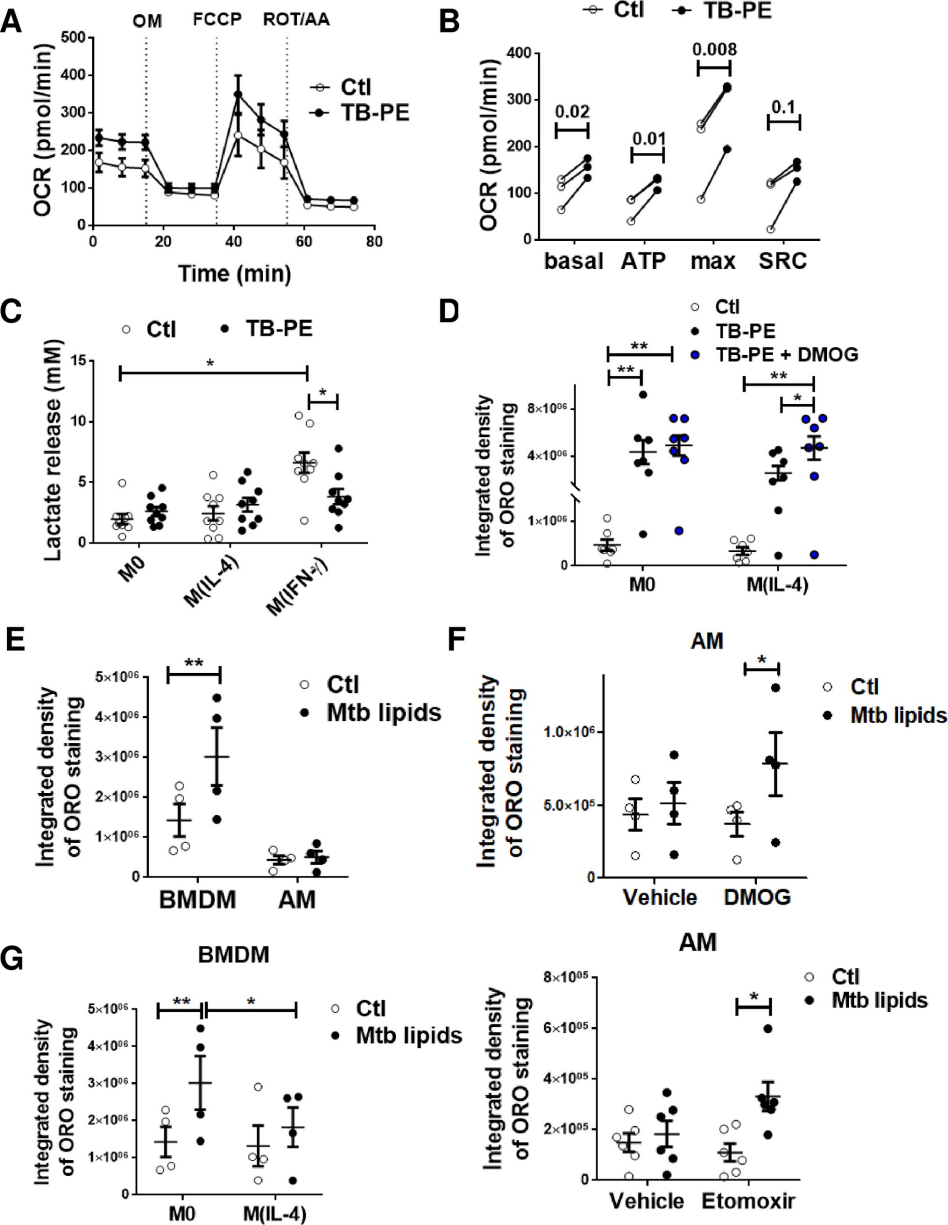
An oxidative metabolism is associated with less accumulation of LBs. **(A-B)** Mitochondrial respiration in M(IL-4) cells exposed or not to TB-PE for 24 h. **(A)** Changes in oxygen consumption rate (OCR) in response to sequential injections of oligomycin (OM), carbonyl cyanide 4-(trifluoromethoxy)phenylhydrazone (FCCP), and rotenone (ROT) + antimycin A (AA) were measured by using an extracellular flux analyzer. Result showed was one representative from three independent experiments. **(B)** Calculated basal respiration, ATP production, maximal respiration and spare respiratory capacity are plotted in bar graphs. Values are expressed as means of triplicates and three independent experiments are shown. Paired t-tests were applied. **(C)** Lactate release by M0, M(IL-4) and M(IFN-γ) cells treated or not with TB-PE. M(IFN-γ) cells serves as positive control. Values are expressed as means ± SEM of nine independent experiments. Friedman test followed by Dunn’s Multiple Comparison Test: ***p<0.001 for M(IFN-γ) vs Ctl. **(D)** Integrated density of ORO staining of M0 and M(IL-4) macrophages treated with TB-PE in the presence or not of DMOG. Values are expressed as means ± SEM of 6 independent experiments, considering five microphotographs per experiment. Friedman test followed by Dunn’s Multiple Comparison Test: *p<0.05; for experimental condition vs Ctl or as depicted by lines. **(E)** Integrated density of ORO staining of murine bone marrow derived macrophages (BMDM) and alveolar macrophages (AM) treated with a total lipids’ preparation from Mtb (Mtb lipids). **(F)** Integrated density of ORO staining of murine AM treated or not with Mtb lipids in the presence of DMOG (upper panel, n = 4) or Etomoxir (lower panel, n = 6). Values are expressed as means ± SEM of seven independent experiments, considering five microphotographs per experiment. **(G)** Murine bone marrow derived macrophages (BMDM) were left untreated (M0) or polarized with IL-4 (M(IL-4)) for 48 h, treated with Mtb lipids for further 24 h, and ORO-stained for quantification of LBs accumulation. Values are expressed as means ± SEM of four independent experiments, considering five microphotographs per experiment. Friedman test followed by Dunn’s Multiple Comparison Test: *p<0.05; **p<0.01 as depicted by lines.

Since M(IL-4) macrophages oxidize fatty acids to fuel the OXPHOS pathway, we hypothesized that their reluctance to accumulate fatty acids and become FM could be associated to a specific metabolic state. First, we demonstrated that the M(IL-4) phenotype was still conserved upon TB-PE treatment, as judged by the expression of CD209, CD200R, and pSTAT6 (**S5C and S5D Fig**). Next, we evaluated the effect of TB-PE treatment on a key function associated to the M(IL-4) profile, such as the uptake of apoptotic polymorphonuclear leukocytes, *i*.*e*. efferocytosis. As observed in **S5E Fig**, TB-PE-treated M(IL-4) cells were even more efficient at efferocytosis than untreated-M(IL-4) cells. As IL-4 levels in TB-PE were undetectable by ELISA (less than 6 pg/ml), the observed effect could not be due to IL-4 within TB-PE. In agreement with this, M(IL-10) or M(IFN-γ) macrophages did not show detectable expression of the phosphorylated form of STAT6 in the presence of TB-PE (**S5D Fig**). Hence, important features associated with alternative activation by IL-4 were not impaired by TB-PE treatment. Moreover, mitochondrial respiration is associated with M(IL-4) macrophages [22]. In this regard, we found a higher consumption of oxygen associated to mitochondrial respiration in TB-PE-treated M(IL-4) macrophages in comparison to untreated cells (**Fig 5A and 5B**). We also measured the release of lactate as evidence of the activation of the aerobic glycolytic pathway, and found that lactate release was lower in M(IL-4) cells (regardless TB-PE treatment) compared to M(IFN-γ) cells, which are known to be glycolytic cells (**Fig 5C** and [27]). Interestingly, TB-PE treatment decreased lactate secretion by M(IFN-γ) macrophages. The glycolytic pathway is highly regulated by HIF-1α activity [12,[12,28]]. In fact, the stabilization of HIF-1α expression resulted in both the upregulation of glycolysis and the suppression of FAO [29]. For this reason, we treated M0 and M(IL-4) with dimethyloxaloylglycine (DMOG), which leads to HIF-1α stabilization [30], and evaluated its impact on FM formation induced by TB-PE. As shown in **Fig 5D and S6A and S6B Fig**, an increase in LBs accumulation was observed after HIF-1α stabilization. Importantly, when we looked at murine alveolar macrophages (AM), which are known to be committed to a FAO and OXPHOS pathway [19], there was no LBs accumulation detected in these cells when exposed to mycobacterial lipids in comparison to bone marrow-derived macrophages (BMDM) (**Fig 5E**). By contrast, when we exposed AM to either a HIF-1α potentiator (DMOG) or a FAO inhibitor (Etomoxir), they became foamy (**Fig 5F and S6C Fig**). Of note, murine IL-4-activated BMDM were less prone to become foamy upon mycobacterial lipid exposure compared to M0 macrophages (**Fig 5G and S6D Fig**). Finally, we also assessed the FM phenotype in human AM isolated from patients undergoing bronchoscopy for clinical reasons unrelated to respiratory infection disease. While there was a high variability of ORO staining in AM from different patients, treatment with either etomoxir or DMOG exacerbated FM formation in these cells (**S6E Fig**). These results clearly demonstrate that an oxidative metabolic state in alternatively activated macrophages is associated with less LBs accumulation and FM formation.

### Mtb infection overrides the metabolic program of M(IL-4) macrophages to induce FM formation

**Fig 6:**
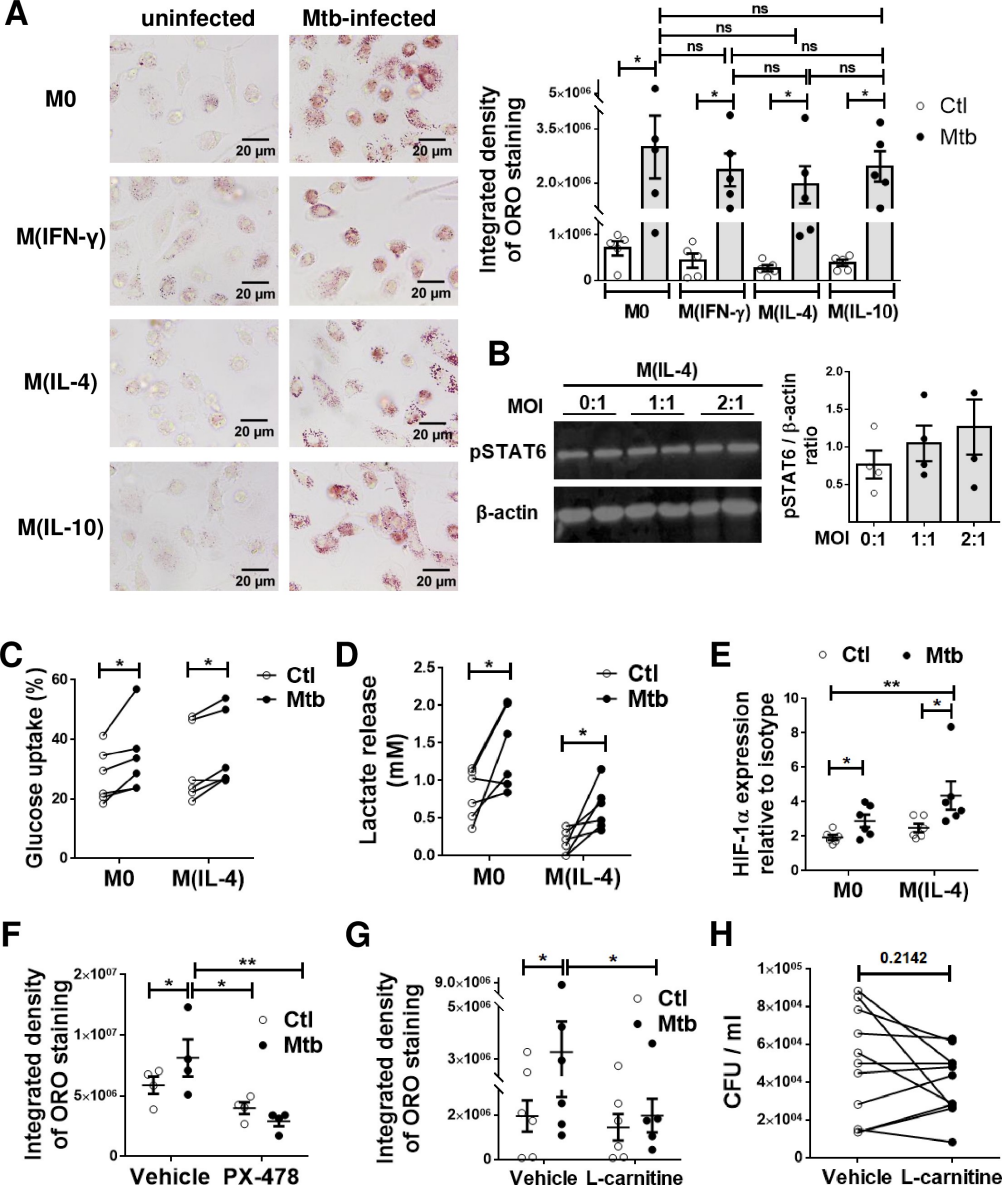
M(IL-4) cells are metabolically reprogrammed and become foamy upon Mtb infection. **(A)** ORO staining of M0, M(IFN-γ), M(IL-4) and M(IL-10) macrophages infected or not with Mtb. Representative images are shown in left panels (40× magnification) and the integrated density of ORO staining is shown in right panels. Values are expressed as means ± SEM of five independent experiments, considering five microphotographs per experiment. **(B)** Analysis of pSTAT6 and β-actin protein expression level by Western Blot and quantification (n = 4) in M(IL-4) cells infected or not with different multiplicity of Mtb infection. **(C-F)** M0 and M(IL-4) macrophages were infected or not with Mtb and the following parameters were measured: **(C)**, glucose consumption (n = 6), **(D)** lactate release (n = 6) and, **(E)** HIF-1α expression (n = 6). **(F-G)** ORO staining of M(IL-4) macrophages infected with Mtb and treated either with PX-478, a selective HIF-1α inhibitor **(F)** or with L-carnitine, a FAO enhancer **(G)**. Values are expressed as means ± SEM of 6 independent experiments, considering five microphotographs per experiment. Friedman test followed by Dunn’s Multiple Comparison Test: *p<0.05; for experimental condition vs Ctl or as depicted by lines. **(H)** Intracellular colony forming units determined of M(IL-4) macrophages infected with Mtb and treated with L-carnitine at day 3 post infection.

As Mtb infection drives FM differentiation [2,31,32], we next investigated whether the different activation profiles of macrophages differ in their ability to become FM when challenged directly with the bacillus. To accomplish this, M0, M(IFN-γ), M(IL-4) and M(IL-10) were infected (or not) with Mtb, and the accumulation of LBs was evaluated at 24 h post-infection. We confirmed that Mtb infection promoted LBs accumulation at comparable levels in all different activation programs, including M(IL-4) macrophages (**Fig 6A**). Since we observed that STAT6 activation mediates the inhibition of FM formation in M(IL-4) macrophages, we assessed whether Mtb infection resulted in a reduction in STAT6 phosphorylation in these cells. However, pSTAT6 was not reduced after Mtb infection of M(IL-4) cells **(Fig 6B)**, suggesting that Mtb targets another molecule downstream of this transcription factor. Given the importance of the metabolic state in our model, we inferred that the M2-like metabolism was probably rewired due to infection. In the case of the glycolytic activity, we found that Mtb infection slightly increased glucose uptake and lactate release in both M0 and M(IL-4) cells **(Fig 6C and 6D)**. In addition, the expression levels of HIF-1α were higher in the Mtb-infected M(IL-4) cells compared to uninfected cells **(Fig 6E).** As a proof-of-concept that M(IL-4) cells are metabolically re-programmed by Mtb infection, we assessed the accumulation of LBs in M(IL-4) macrophages in the presence of either a HIF-1α inhibitor (PX-478) or a FAO enhancer (L-carnitine). As expected, both treatments resulted in less accumulation of LBs triggered by Mtb infection in M(IL-4) macrophages (**Fig 6F and 6G and S7 Fig**). However, despite its effect on LBs accumulation, treatment with L-carnitine did not influence the control of bacterial growth compared to untreated M(IL-4) macrophages (**Fig 6H**). Altogether, these findings demonstrate that the modulation of M(IL-4) metabolisms impacts on the acquisition of the foamy phenotype induced by Mtb.

## Discussion

As a chronic inflammatory condition, TB entails the establishment of extensive metabolic reprogramming in both the host and the pathogen. One of the consequences of this metabolic adaptation is FM formation. Since FM have been associated with the mycobacterial persistence and tissue pathology [2,31,33,34], we aimed to determine the impact of different activation/metabolic programs in human macrophages on LBs accumulation. By using the acellular fraction of TB-PE, known to induce FM formation in M0 and M(IL-10) macrophages [6], we demonstrated that STAT6 activation induced either by IL-4 or IL-13 prevents the accumulation of LBs in M(IL-4) macrophages. Compelling evidence from the literature suggest that FM are formed in response to the infection process, but not necessarily as a result of the direct contact with the mycobacteria. Indeed, it was reported that, although most of the bacilli within pulmonary TB lesions was located within the same area as FM, not all FM are infected by the bacteria as detected by Ziehl Neelsen staining [32], suggesting that lipids are also accumulated in uninfected cells within the site of infection. In line with this, several studies demonstrated that mycobacterial cell wall lipid-containing vesicles are released from infected macrophages to neighbouring cells making them foamy [35–37], indicating that factors present in the microenvironment can promote the foamy phenotype in uninfected bystander macrophages [31]. These findings may also explain why tissue damage is high in human TB granulomas where the bacterial density is relatively low [38]. In the *in vitro* context, both infected and uninfected macrophages exhibited a similar elevated lipid droplet content, implying that a paracrine signal may be associated with infection-induced lipid droplet accumulation [39]. Interestingly, the presence of FM in lungs is widely associated with the occurrence of tissue necrosis, thereby opening up the possibility that uninfected macrophages surrounding the necrotic centre within granulomas may differentiate to FM using lipids available from the necrotic cells [40]. All these findings support the relevance of studying the involvement of the TB microenvironment in the formation of FM.

Importantly, we provide *ex vivo* evidence for the refractoriness of M(IL-4/IL-13) macrophages to become foamy by finding a negative correlation between the expression of CD209, a *bona fide* marker in human M(IL-4)-associated, and the numbers of LBs-containing CD14^+^ cells isolated directly from the pleural cavity of TB patients, thus providing physiological relevance to our *in vitro* findings. Since TB-PE have, if any, very few mycobacterial load [41], we consider that the impact of potential *in situ* infection on the macrophage metabolic state, which may then lead M(IL-4) cells to become FM, might be very low. Additionally, while CD206 is also known to be induced by IL-4 and IL-13 [42], we did not find such an association between this marker and the numbers of FM. This can be due to the fact that its expression is also induced upon other activation programs such as M(IL-10), which are demonstrated to express high levels of CD206 [21,43] and prone to synthesize and accumulate LBs [6]. In fact, we have previously demonstrated that FM were formed after TB-PE treatment by increasing the biogenesis of LBs through the up-regulation of ACAT by the IL-10/STAT3 axis [6]. In this study, we observed that the STAT3 pathway is also induced in TB-PE-treated M(IL-4) macrophages resulting in ACAT induction, but as the newly formed LBs are rapidly disrupted in these macrophages, the foamy appearance was reduced drastically. In this regard, we now showed that, while LBs are formed, they are quickly disrupted by an enhanced lipolytic activity induced in M(IL-4) macrophages, and those fatty acids produced through lipolysis are then incorporated into the mitochondria and used for FAO. Based on these results, we argue that IL-10/STAT3 and IL-4/STAT6 axes may operate at different steps of the life cycle of LBs; that is, while the former is responsible for driving the biogenesis of LBs, the latter controls their lipolysis. Aside from this, a possible crosstalk between these two axes may occur through an inhibitory effect of IL-10 signalling on the FAO pathway, resulting in the promotion of LBs accumulation in macrophages. Yet, this hypothesis requires further investigation.

Moreover, we demonstrated that cell populations known to rely on oxidative metabolism are less prone to become FM. This phenomenon is not only specific to human M(IL-4) or M(IL-13) treated with TB-PE, but also to different human or mice macrophage populations (MDM, BMDM and AM), and in response to different stimuli such as TB-PE and Mtb lipids. Hence, our findings are strengthened by previous reports showing that resveratrol, a natural product that enhances mitochondrial metabolism [44], also impairs LBs accumulation in macrophages [45].

Interestingly, the inhibition of lipids oxidation in M(IL-4) macrophages results in diverting the fatty acid fate into triglycerides accumulation, leading these cells to form FM. Although the M2 program was shown to rely on FAO [13–16], the precise role of FAO in driving M2 polarization requires further investigation [46,47]. Herein, we found that the exacerbated FAO activity prevents lipid accumulation and FM formation. Our results are in agreement with previous reports in the field of atherosclerosis, showing that enforcing CPT1a expression can reduce [48], arguing that the induction of FAO in foam cells could be of therapeutic potential. Also, in adipocytes, it has been demonstrated that IL-4 harbors pro-lipolysis capacity by inhibiting adipocyte differentiation and lipid accumulation, as well as by promoting lipolysis in mature cells to decrease lipid deposits [49]. Moreover, supported by our previous report and others, the differentiation of macrophages into FM (prior to infection) renders the cells more susceptible to the intracellular replication of Mtb [6,50,51]. Here, we extended this notion to the M(IL-4) macrophage profile exposed to lipases inhibitors. We have previously shown that FM induced by TB-PE had immunosuppressive properties such as: i) high production of IL-10, ii) low production of TNF-α, iii) poor induction of IFN-γ producing T clones in response to mycobacterial antigens, and iv) more permissiveness to intracellular mycobacterial growth [6]. Along with other reports that associated FM with the persistence of infection [2,32], these findings support the idea that the generation of FM would have a negative impact for mycobacterial control; conversely, the reluctance of macrophages to become foamy mediated by the IL-4/IL-13-STAT6 axis could have positive consequences for the host. Surprisingly, however, the infection with Mtb *per se* promoted the LBs accumulation in M(IL-4) macrophages despite the activation of the IL-4/STAT6 axis. In fact, Mtb may induce the foamy phenotype by hijacking a metabolic pathway downstream to STAT6 activation. In addition, Mtb infection can decrease the lipolytic activity of macrophages at early time points [5,34]. Therefore, we propose that lipolysis modulation is key for determining lipid accumulation in the context of Mtb infection. Of note, although we were able to inhibit FM differentiation upon Mtb infection by fostering the FAO pathway with L-carnitine, the cells were still susceptible to Mtb intracellular growth. This finding may be explained by the fact that macrophages endowed with a dominant FAO metabolic state are characterized by a M2-like profile and a poor microbicidal activity [19]. Therefore, while metabolic reprogramming of lipid-laden FM following Mtb infection represents a promising target for host-directed therapy, caution should be taken about unwanted effects resulting from the modulation of metabolic states such as impairment of microbicidal activity.

Another contribution of this study is the notion that Mtb infection reprograms the metabolic state of M(IL-4) macrophages, leading to FM formation through positive regulation of HIF-1α, without affecting STAT6 phosphorylation. We consider that this mechanism constitutes a strategy of mycobacterial persistence. Indeed, a high lipid content guarantees the survival of the pathogen, and the activation of the IL-4/STAT6 axis is associated with the establishment of a poorly microbicidal profile [9]. Previous reports demonstrated that Mtb infection leads to glycolysis in BMDM [52,53], in lungs of infected mice [54], and in pulmonary granulomas from patients with active TB [55]. Here, we showed that Mtb infection leads to HIF-1α activation and lactate release in M(IL-4) macrophages. Of note, Mtb infection induces the increase of HIF-1α expression in IFN-γ-activated macrophages (M1), which is essential for IFN-γ-dependent control of infection [56]. It is also important to highlight that Mtb reprograms the metabolic state of M(IL-4) macrophages without affecting STAT6 activation. This means that the pro-inflammatory program potentially established by HIF-1α activation may also be modulated by the inhibitory signals driven by the IL-4/STAT6 pathway. Hence, we speculate that foamy M(IL-4) macrophages can bear high bacillary loads despite HIF-1α activation, especially considering that LBs were described as a secure niche for mycobacteria conferring protection even in the presence of bactericidal mechanisms, such as respiratory burst [5]. Moreover, our findings agree with a recent article by Zhang and colleagues in which they demonstrated that a mouse model with a targeted deficiency in the macrophage compartment for the E3 ligase von Hippel–Lindau protein (VHL), an enzyme that keeps HIF-1α at a low level *via* ubiquitination followed by proteasomal degradation, exhibited an enhanced glycolytic metabolic state in AM. Although not highlighted by the authors, these AM displayed a foamy phenotype unlike those from control animals [57], arguing that the uncontrolled activation of HIF-1α contributes to FM formation. In line with this, it was recently described that HIF-1α activation promotes the survival of infected FM during late stages of Mtb infection [58]; another study demonstrated HIF-1α activation mediated by IFN-γ contributes to the formation of LBs in Mtb-infected macrophages [59]. An important enhancer of HIF-1α activity is the mammalian target of rapamycin (mTOR) complex 1 (mTORC1). In monocytes/macrophages, the upregulation of HIF-1α expression after Mtb infection is TLR2-dependent, and is partly mediated by the activation of the protein kinase B (AKT)-mTOR pathway [60]. Thus, the HIF-1α-dependent metabolism may be linked to the formation of FM *via* mTOR activation. In this regard, triglyceride accumulation in human macrophages infected with Mtb was shown to be mediated by mTORC1 [39]. At least two hypotheses can explain how HIF-1α activation contributes to FM formation: 1) by suppressing FAO, and/or 2) by inducing endogenous fatty acid synthesis. Although in this study we did not address the endogenous synthesis of fatty acids of infected macrophages, we were able to demonstrate that both the inhibition of HIF-1α activity and the exacerbation of FAO partially decrease the acquisition of the foamy phenotype in M(IL-4) macrophages infected by Mtb. A possible link between the increase in HIF-1α and the decrease in FAO was demonstrated in another experimental model, where both HIF-1α and HIF-2α mediate the accumulation of lipids in hepatocytes by reducing PGC-1α-mediated FAO [61].

It is worth mentioning that FM are present in several inflammatory diseases, such as atherosclerosis and chronic infectious diseases, among others. Thus, the accumulation of LBs within macrophages is not exclusive for TB. In addition, Guerrini *et al*. demonstrated that, unlike the cholesterol-laden cells of atherosclerosis, foam cells in tuberculous lung lesions accumulate triglycerides [39], indicating that different adipogenic mechanisms may operate even within the TB context. Indeed, while the high levels of cholesterol present in TB-PE may drive LBs formation in exposed cells [6], triglycerides may alternatively do so in Mtb-infected cells.

Collectively, our study sheds light on the remarkable capacity of Mtb to rewire the metabolic state of macrophages to induce FM formation, including the reluctant alternatively activated program established by IL-4 and IL-13. This is important because, in the context of TB, there is a frequent shift from M1 to M2 program of macrophage activation at sites of inflammation that is associated with the adaptive immune transition from acute to chronic phases; this shift is thought be critical for inflammation resolution and tissue repair at infection sites [62]. As alternatively activated macrophages promote tissue healing, repair and growth, our findings also demonstrate these cells, thanks to their FAO metabolic state, withstand tissue environmental conditions shaped by TB disease (*e*.*g*., TB-PE) to refrain their potential differentiation into FM.

Macrophages are the most abundant cells in TB granulomas, indeed temporal and spatial changes in their polarization have been described [63,64]. M1 macrophages are found in the central granuloma regions that contains Mtb-infected macrophages, which contrasts the peripheral granuloma regions containing M2 macrophages [63,65]. FM are found close to the core of the granulomas in contact with Mtb [2,32], and thus they are likely to express M1 markers. Also, the hypoxic environment found within the core of granulomas probably favors HIF-1α activation in macrophages. Even when M1 markers are identified in the sections enriched with FM, the activation program is likely to be influenced by Mtb infection since a shift from M1 to M2 polarization is known to take place [66,67]. If so, our study predicts that Mtb infection will exert a HIF-1α-dependent dominant effect in the metabolic program of alternatively activated macrophages to induce FM. Therefore, we infer the interplay between STAT6 and HIF-1α signaling pathways in macrophages will constitute a novel paradigm for FM formation and TB pathogenesis.

## Materials and methods

### Ethics statement

The research was carried out in accordance with the Declaration of Helsinki (2013) of the World Medical Association and was approved by the Ethics Committees of the Hospital F.J Muñiz and the Academia Nacional de Medicina de Buenos Aires (protocol number: NIN-1671-12, renewed in 2018; and 12554/17/X, respectively). Written informed consent was obtained before sample collection.

### Chemical reagents

DMSO, AS1517499, Etomoxir, L-carnitine, and Oil red O were obtained from Sigma-Aldrich (St. Louis, MO, USA); Lalistat was purchased from Bio-techne (Abingdon, UK), Tetrahydrolipstatin (Orlistat) from Parafarm (Buenos Aires, Argentina), PX-478 2HCL from Selleck Chemicals (Houston, TX, USA) and DMOG from Santa Cruz, Biotechnology (Palo Alto, CA, USA).

### Bacterial strain and antigens

Mtb H37Rv strain was grown at 37°C in Middlebrook 7H9 medium supplemented with 10% albumin-dextrose-catalase (both from Becton Dickinson, NJ, United States) and 0.05% Tween-80 (Sigma-Aldrich). The Mtb γ-irradiated H37Rv strain (NR-49098) and its total lipids´ preparation (NR-14837) were obtained from BEI Resource, Manassas, VA, USA. The RFP-expressing Mtb strain was gently provided by Dr. Fabiana Bigi (INTA, Castelar, Argentina).

### Preparation of human monocyte-derived macrophages

Buffy coats from healthy donors were prepared at Centro Regional de Hemoterapia Garrahan (Buenos Aires, Argentina) according to institutional guidelines (resolution number CEIANM-664/07). Informed consent was obtained from each donor before blood collection. Monocytes were purified by centrifugation on a discontinuous Percoll gradient (Amersham, Little Chalfont, UK) as previously described [6]. After that, monocytes were allowed to adhere to 24-well plates at 5x10^5^ cells/well for 1 h at 37°C in warm RPMI-1640 medium (ThermoFisher Scientific, Waltham, MA, USA). The medium was then supplemented to a final concentration of 10% Fetal Bovine Serum (FBS, Sigma-Aldrich) and human recombinant Macrophage Colony-Stimulating Factor (M-CSF, Peprotech, Rocky Hill, NJ, USA) at 10 ng/ml. Cells were allowed to differentiate for 5–7 days. Thereafter, macrophage activation was induced by adding either IL-4 (20 ng/ml, Biolegend, San Diego, CA, USA), IL-13 (20 ng/ml, Inmmunotools, Friesoythe, Germany), IL-10 (10 ng/ml, Peprotech), or IFN-γ (10 ng/ml, Peprotech) for further 48 h, resulting in M(IL-4), M(IL-13), M(IL-10) and M(IFN-γ) respectively.

### Preparation of pleural effusion pools

Pleural effusions (PE) were obtained by therapeutic thoracentesis by physicians at the Hospital F.J Muñiz (Buenos Aires, Argentina). Individual TB-PE samples were used for correlation analysis. A group of samples (n = 10) were pooled and used for *in vitro* assays to treat macrophages. The diagnosis of TB pleurisy was based on a positive Ziehl-Neelsen stain or Lowestein–Jensen culture from PE and/or histopathology of pleural biopsy and was further confirmed by an Mtb-induced IFN-γ response and an adenosine deaminase-positive test [68]. Exclusion criteria included a positive HIV test, and the presence of concurrent infectious diseases or non-infectious conditions (cancer, diabetes, or steroid therapy). None of the patients had multidrug-resistant TB. PE samples derived from patients with pleural transudates secondary to heart failure (HF-PE, n = 5) were employed to prepare a second pool of PE, used as control of non-infectious inflammatory PE. The PE were collected in heparin tubes and centrifuged at 300 g for 10 min at room temperature without brake. The cell-free supernatant was transferred into new plastic tubes, further centrifuged at 12000 g for 10 min and aliquots were stored at -80°C. The pools were decomplemented at 56°C for 30 min and filtered by 0.22 μm in order to remove any remaining debris or residual bacteria.

### Foamy macrophage induction

Activated macrophages were treated with or without 20% v/v of PE, 10 μg/ml of Mtb lipids (BEI resources) or infected with Mtb (MOI 2:1) for 24 h. When indicated, cells were pre-incubated with either STAT6 inhibitor AS1517499 (100 nM, Sigma-Aldrich) for 30 min prior to IL-4 and TB-PE addition, orlistat (100 μM) and lalistat (10μM) for further 1 h after TB-PE incubation, etomoxir (3 and 10μM) for 30 min prior to TB-PE addition, DMOG (100μM) during TB-PE incubation and L-carnitine (1 and 10mM) for 48 h prior and during TB-PE addition. Foam cell formation was followed by Oil Red O (ORO) staining (Sigma-Aldrich) as previously described [6,69] at 37°C for 1–5 min and washed with water 3 times. For the visualization of the lipid bodies, slides were prepared using the aqueous mounting medium Poly-Mount (Polysciences Inc, PA, USA), observed via light microscope (Leica) and finally photographed using the Leica Application Suite software. For the determination of LBs size and numbers, images of ORO-stained cells were quantified with the ImageJ “analyze particles” function in thresholded images, with size (square pixel) settings from 0.1 to 100 and circularity from 0 to 1. For quantification, 10–20 cells of random fields (100x magnification) per donor and per condition were analyzed.

### Phenotypic characterization by flow cytometry

Macrophages were stained for 40 min at 4°C with fluorophore-conjugated mAb FITC-anti-CD36 (clone 5–271, Biolegend), or PE-anti-CD209 (clone 120507, R&D Systems, Minneapolis, MN, USA), and in parallel, with the corresponding isotype control antibody. After staining, the cells were washed with PBS 1X, centrifuged and analyzed by flow cytometry using a FACSCalibur cytometer (BD Biosciences, San Jose, CA, USA) The monocyte-macrophage population was gated according to its Forward Scatter and Size Scatter properties. The median fluorescence intensity (MFI) was analyzed using FCS Express V3 software (De Novo Software, Los Angeles, CA, USA).

### Western blots

Protein samples were subjected to 10% SDS-PAGE and the proteins were then electrophoretically transferred to Hybond-ECL nitrocellulose membranes (GE Healthcare, Amersham, UK). After blocking with 1% bovine serum albumin (BSA, DSF Lab, Córdoba, Argentina), the membrane was incubated with anti-human ACAT (1:200 dilution, SOAT; Santa Cruz) or anti-human pY641-STAT6 (1:200 dilution, Cell Signaling Technology, Danvers, MA, USA, clone D8S9Y) antibodies overnight at 4°C. After extensive washing, blots were incubated with HRP-conjugated goat anti-rabbit IgG (1:5000 dilution; Santa Cruz) antibodies for 1 h at room temperature. Immunoreactivity was detected using ECL Western Blotting Substrate (Pierce, ThermoFisher). Protein bands were visualized using Kodak Medical X-Ray General Purpose Film. For internal loading controls, membranes were stripped and then reprobed with anti-β-actin (1:2000 dilution; ThermoFisher, clone AC-15) or anti-STAT6 (1:1000 dilution; Cell Signaling Technology, clone D3H4) antibodies. Results from Western blot were analyzed by densitometric analysis (Image J software).

### Determination of metabolites

Glycerol release was determined following Garland and Randle`s protocol [70] with some modifications. Briefly, cell-culture medium was removed and replaced by PBS containing fatty-acid free 2% BSA. Macrophages were incubated for 6 h at 37°C and supernatants were collected and assessed with the enzymatic kit TG Color GPO/PAP AA (Wiener, Buenos Aires, Argentina) according to the manufacturer’s instructions. In all cases, the absorbance was read using a Biochrom Asys UVM 340 Microplate Reader microplate reader and software.

### Determination of fatty acids uptake

Macrophages were incubated with 2.4 μM BODIPY 558/568 C12 (Red C12, Life Technologies, Waltham, MA, USA) in PBS containing fatty-acid free 0.1% BSA, for 1 min at 37°C. Cells were immediately washed with cold PBS containing 0.2% BSA and run through the FACS machine using FL2 to determine fatty acids uptake.

### Measurement of cell respiration with Seahorse flux analyzer

Real-time oxygen consumption rate (OCR) in macrophages was determined with an XFp Extracellular Flux Analyzer (Seahorse Bioscience). The assay was performed in XF Assay Modified DMEM using 1.6x10^5^ cells/well and 3 wells *per* condition. Three consecutive measurements were performed under basal conditions and after the sequential addition of the following electron transport chain inhibitors: 3 μM oligomycin (OM), 1 μM carbonyl cyanide 4-(trifluoromethoxy)phenylhydrazone (FCCP), 0.5 μM rotenone (ROT) and 0.5 μM antimycin (AA). Basal respiration was calculated as the last measurement before addition of OM minus the non-mitochondrial respiration (minimum rate measurement after ROT/AA). Estimated ATP production designates the last measurement before addition of OM minus the minimum rate after OM. Maximal respiration rate (max) was defined as the OCR after addition of OM and FCCP. Spare respiration capacity (SRC) was defined as the difference between max and basal respiration.

### Quantitative RT-PCR

Total RNA was extracted with Trizol reagent (Sigma-Aldrich) and cDNA was reverse transcribed using the Moloney murine leukemia virus reverse transcriptase and random hexamer oligonucleotides for priming (Life Technologies). The expression of CPT1 was determined using PCR SYBR Green sequence detection system (Eurogentec, Seraing, Belgium) and the CFX Connect Real-Time PCR Detection System (Bio-Rad, Hercules, CA, USA). Gene transcript numbers were standardized and adjusted relative to eukaryotic translation elongation factor 1 alpha 1 (EeF1A1) transcripts. Gene expression was quantified using the ΔΔCt method.

### Transmission electron microscopy (TEM)

M0 and M(IL-4) cells exposed to TB-PE were prepared for TEM analysis. For this purpose, cells were fixed in 2.5% glutaraldehyde / 2% paraformaldehyde (PFA, EMS, Delta-Microscopies, France) dissolved in 0.1 M Sorensen buffer (pH 7.2) during 2 h at room temperature and then they were preserved in 1% PFA dissolved in Sorensen buffer. Adherent cells were treated for 1 h with 1% aqueous uranyl acetate then dehydrated in a graded ethanol series and embedded in Epon. Sections were cut on a Leica Ultracut microtome and ultrathin sections were mounted on 200 mesh onto Formvar carbon-coated copper grids. Finally, thin sections were stained with 1% uranyl acetate and lead citrate and examined with a transmission electron microscope (Jeol JEM-1400) at 80 kV. Images were acquired using a digital camera (Gatan Orius). For the determination of LBs size and number, TEM images were quantified with the ImageJ “analyze particles” plugins in thresholded images, with size (μm^2^) settings from 0.01 to 1 and circularity from 0.3 to 1. For quantification, 8–10 cells of random fields (1000x magnification) per condition were analyzed.

### Infection of human macrophages with Mtb

Infections were performed in the biosafety level 3 (BSL-3) laboratory at the Unidad Operativa Centro de Contención Biológica (UOCCB), ANLIS-MALBRAN (Buenos Aires), according to the biosafety institutional guidelines. Macrophages seeded on glass coverslips within a 24-well tissue culture plate (Costar) at a density of 5x10^5^ cells/ml were infected with Mtb H37Rv strain at a MOI of 2:1 during 1 h at 37°C. When indicated, PX-478 (100 μM) was added or not 1 h prior to Mtb infection and renewed in fresh complete media after cell-washing. Then, extracellular bacteria were removed gently by washing with pre-warmed PBS, and cells were cultured in RPMI-1640 medium supplemented with 10% FBS and gentamicin (50 μg/ml) for 24 h. In some experiments, L-carnitine (10 mM) was added or not after removing the extracellular bacteria. The glass coverslips were fixed with 4% PFA and stained with ORO, as was previously described.

### Measurement of bacterial intracellular growth in macrophages by CFU assay

Macrophages exposed (or not) to TB-PE, were infected with H37Rv Mtb strain at a MOI of 0.2 bacteria/cell in triplicates. After 4 h, extracellular bacteria were removed by gently washing four times with pre-warmed PBS. At different time points cells were lysed in 1% Triton X-100 in Middlebrook 7H9 broth. Serial dilutions of the lysates were plated in triplicate, onto 7H11-Oleic Albumin Dextrose Catalase (OADC, Becton Dickinson) agar medium for CFU scoring 21 days later.

### Visualization and quantification of Mtb infection

Macrophages seeded on glass coverslips within a 24-well tissue culture plate (Costar) at a density of 5×10^5^ cells/ml were infected with the red fluorescent protein (RFP) expressing Mtb CDC1551 strain at a MOI of 5:1 during 2 h at 37°C. Then, extracellular bacteria were removed gently by washing with pre-warmed PBS, and cells were cultured in RPMI-1640 medium supplemented with 10% FBS for 48 h. The glass coverslips were fixed with 4% PFA and stained with BODIPY 493/503 (Life Technologies). Finally, slides were mounted and visualized with a FluoView FV1000 confocal microscope (Olympus, Tokyo, Japan) equipped with a Plapon 60X/NA1.42 objective, and then analyzed with the software ImageJ-Fiji. We measured the occupied area with RFP-Mtb (expressed as Raw Integrated Density) per cell in z-stacks from confocal laser scanning microscopy images. Individual cells were defined by BODIPY-stained cellular membranes which allow us to define the region of interests for quantification. For quantification 80–100 cells of random fields per donor per condition were analyzed.

### Mice

BALB/c male mice (8–12 weeks old) were used. Animals were bred and housed in accordance with the guidelines established by the Institutional Animal Care and Use Committee of Institute of Experimental Medicine (IMEX)-CONICET-ANM. All animal procedures were shaped to the principles set forth in the Guide for the Care and Use of Laboratory Animals [71].

### Culture of BMDMs and AMs

Femurs and tibia from mice were removed after euthanasia and the bones were flushed with RPMI-1640 medium by using syringes and 25-gauge needles. The cellular suspension was centrifuged, and the red blood cells were removed by hemolysis with deionized water. The BMDMs were obtained by culturing the cells with RPMI-1640 medium containing L-glutamine, pyruvate, streptomycin, penicillin and β-mercaptoethanol (all from Sigma-Aldrich), 10% FBS and 20 ng/ml of murine recombinant M-CSF (Biolegend) at 37°C in a humidified incubator for 7–8 days. Differentiated BMDMs were re-plated on glass coverslips within 24-well tissue culture plates in complete medium and polarized or not with murine recombinant IL-4 (20 ng/ml, Miltenyi Biotec, Auburn, CA, USA) for 48 h. AMs were harvested via bronchoalveolar lavage and plated on glass coverslips within a 24-well tissue culture plate in complete medium. Finally, BMDMs and AMs were stimulated with Mtb lipids (10 μg/ml) in the presence or not of DMOG (200μM) or etomoxir (3 μM) for 24 h and LBs accumulation was determined.

### Statistical analysis

Values are presented as means and SEM of a number of independent experiments or as indicated in figure legends. Independent experiments are defined as those performed with macrophages derived from monocytes isolated independently from different donors. For non-parametric paired data, comparisons were made using the Friedman test followed by Dunn's Multiple Comparison Test or by two-tailed Wilcoxon Signed Rank, depending on the numbers of experimental conditions to be compared. For parametric unpaired data, comparisons between two data sets were made by Mann Whitney test. For the analysis of the OCR measurements, the paired t-test was applied. Correlation analyses were determined using the Spearman’s rank test. For all statistical comparisons, a p value <0.05 was considered significant.

## Supporting information

S1 Fig Characterization of cell-surface and molecular markers’ expression in macrophages. Macrophages were stained with fluorophore-conjugated mAbs PE-anti-CD163 (clone GHI/61), APC-anti-MerTK (clone 590H11G1E3), FITC-anti-CD206 (clone C068C2), PerCP.Cy5.5-anti-CD86 (clone 374216) (all from Biolegend), PE-anti-CD209 (clone, R&D Systems), or FITC-anti-HLA-DR (clone G46-6, BD Biosciences), and in parallel, with the corresponding isotype control antibody. The monocyte-macrophage population was gated according to its Forward Scatter and Size Scatter properties. **(A)** Median fluorescence intensity (MFI) of CD206, CD209, CD163, MerTK, CD86, and HLA-DR measured by flow cytometry on M(IL-4), M(IL-10) and M(IFN-γ) macrophages. Representative histograms and values of ten independent experiments are shown. Friedman test followed by Dunn’s Multiple Comparison Test: **p<0.01; ***p<0.001; as depicted by lines. **(B)** Analysis of pSTAT6 (anti-human pY641-STAT6, 1:200 dilution, clone D8S9Y), STAT6 (anti-STAT6, 1:1000 dilution; clone D3H4), pSTAT3 (anti-human pY705-STAT3, 1:1000 dilution, clone D3A7), STAT3 (anti-STAT3, 1:1000 dilution; clone D1A5), pSTAT1 (anti-human pY701-STAT1, 1:1000 dilution, clone D4A7), STAT1 anti-STAT1, 1/500 dilution), all from Cell Signaling Technology, and β-actin protein (1:2000 dilution; ThermoFisher, clone AC-15) expression level by Western Blot and quantification in M(IL-4), M(IL-10) and M(IFN-γ) macrophages.
(TIF)

S2 Fig STAT-6 activation induced by IL-4 and/or IL-13 prevents the formation of FM. **(A)** MFI of CD209, CD206, and CD200R measured by flow cytometry on M0, M(IL-4), and macrophages polarized with different amounts of recombinant IL-13. Left panel represents the analysis of pSTAT6 and β-actin protein expression level M0, M(IL-4), and M(IL-13) by Western Blot **(B-C)** Representative images of ORO staining of macrophages polarized with either different doses of recombinant IL-4 (B) or IL-13 (C) for 48 h and exposed to TB-PE for further 24 h (40x magnification). **(D)** Cell viability of M(IL-4) macrophages exposed to different amounts of AS1517499 or vehicle. **(E)** Analysis of pSTAT6, STAT6, and β-actin protein expression level by Western Blot (right panel) and quantifications (left panels, n = 4) in M0 and M(IL-4) macrophages exposed to AS1517499 or vehicle. Wilcoxon signed rank test: *p<0.05 as depicted by lines.
(TIF)

S3 Fig Association between the phenotype of CD14^+^ cells and the accumulation of LBs in macrophages. **(A)** Correlation study between the MFI of CD206, HLA-DR, CD86, CD163, and MerTK cell-surface expression in CD14^+^ cells from TB pleural cavity and the percentage of lipid-laden CD14^+^ cells within the pleural fluids mononuclear cells (PFMC) (n = 16) found in individual preparations of TB-PE. Spearman’s rank test. **(B)** Analysis of pSTAT3, STAT3 and β-actin protein levels by Western Blot (left panel) and quantifications (right panel, n = 4) in M0 and M(IL-4) macrophages treated or not with TB-PE for 24 h (n = 4). Wilcoxon signed rank test: *p<0.05.
(TIF)

S4 Fig Inhibition of lipolysis in M(IL-4) macrophages restores FM phenotype and impairs the control of Mtb intracellular growth. **(A)** Glycerol release by M0, M(IL-4) and M(IL-13) macrophages (left panel, n = 6). **(B)** Cell viability of M(IL-4) macrophages exposed to Orlistat (upper panel, n = 4) and Lalistat (lower panel, n = 4). **(C)** Glycerol release by M0 and M(IL-4) macrophages exposed or not to either Orlistat (left panel, n = 6) or Lalistat (right panel, n = 4). **(D-E)** M(IL-4) macrophages infected with RFP-Mtb and stained with BODIPY 493/503 at 2 h and 48 h post-infection. **(D)** BODIPY intensity (left panel) and area occupied by RFP-Mtb (right panel) per cell in z-stacks from confocal laser scanning microscopy images at 2 h post-infection. Each determination represents individual cells of one donor. One way-ANOVA followed by Bonferroni test: ***p<0.001. **(E)** Area with RFP-Mtb per cell in z-stacks from confocal laser scanning microscopy images. Values are expressed as means of 80–100 cells in four independent experiments. Friedman followed by Dunn’s Multiple Comparison Test: *p<0.05 as depicted by lines. **(F)** M(IL-4) macrophages were treated or not with TB-PE in the presence or not of Orlistat for 24 h, washed and infected with Mtb (MOI = 5). After 48 h, cells were stained with the fixable vital dye eFluor 780. The percentages of non-viable cells are shown. Mean +/- SEM, N = 4.
(TIF)

S5 Fig Features associated with alternative activation of macrophages were not impaired by TB-PE treatment. **(A)** Cell viability of M(IL-4) macrophages exposed to different amounts of Etomoxir (n = 4). **(B)** Cell viability of M0 macrophages exposed to different amounts of L-carnitine (n = 8). **(C)** MFI of CD209, and CD200R measured by flow cytometry on M(IL-4) treated or not with TB-PE. **(D)** Analysis of pSTAT6, STAT6, and β-actin protein expression level by Western Blot (left panel) in M(IFN-γ), M(IL-4), and M(IL-10) macrophages treated or not with TB-PE. Quantifications in M(IL-4) cells (right panels, n = 4). Wilcoxon signed rank test: *p<0.05. **(E)** CFSE-labelled apoptotic neutrophils (PMN) were cocultured with M(IL-4) macrophages treated or not with TB-PE for 1 h, washed stringently to remove free PMN, and the percentage of macrophages positive for CFSE was assessed. Representative dot blots showing CFSE labeling of apoptotic PMN, M(IL-4), and cocultures are shown. Blue gates represent mainly CFSE labeling of apoptotic PMN while red gates comprise mainly macrophages. Lower panels: Annexin V positive vs propidium iodide negative of apoptotic PMN (left panel) and percentages of M(IL-4) treated or not with TB-PE macrophages ingesting apoptotic PMN (right panel). M(IFN-γ) macrophages were also tested for comparison.
(TIF)

S6 Fig Human and murine macrophages known to have an oxidative metabolism accumulate less LBs. **(A)** Human macrophages were left untreated (M0) or polarized with IL-4 (M(IL-4)) for 48h, treated or not with the acellular fraction of TB pleural effusions (TB-PE) in the presence or not of DMOG (100 μM) for 24 h and then stained with Oil Red O (ORO). Representative images are shown (40× magnification). **(B)** Cell viability of M0 macrophages exposed to different amounts of DMOG. **(C)** Left panel: Representative images (40× magnification) of ORO staining of murine AM treated or not with Mtb lipids in the presence of either DMOG (200 μM) or Etomoxir (3 μM). Right panel: Lactate release and glucose consumption by AM treated or not with Mtb lipids in the presence of DMOG for 24 h (n = 3). One-way ANOVA followed by Bonferroni's Multiple Comparison Test: *p<0.05; **p<0.01, as depicted by lines**. (D)** Representative images (40× magnification) of ORO staining of murine bone marrow derived macrophages murine (BMDM) unpolarized or polarized towards M(IL-4), exposed or not to Mtb lipids. **(E)** Leftovers of bronchoalveolar lavages from unidentified patients undergoing bronchoscopy for clinical reasons unrelated to pulmonary infections were collected, according to the protocol approved by the Ethics Committees of the Hospital F.J Muñiz. Bronchoalveolar lavage fluid was centrifuged at 1,400 rpm for 10 minutes. Cells were resuspended at 5 × 10^5^ cells / ml in RPMI 1640 culture media supplemented with FBS, 100 U/ml penicillin, and 100 μg/ml streptomycin. Adherence purification of AM was performed; nonadherent cells were removed by washing after 1 h. Human alveolar macrophages isolated from 3 different donors were treated or not with etomoxir (3 μM) or DMOG (200 nM) for 1 h, prior to Mtb lipids stimulation for further 24 h and then stained with Oil Red O (ORO). Left panel: Representative images (40× magnification), right panel: quantification of ORO staining. Each dot represents the mean of five or six micrograph per condition. Kruskal-Wallis test followed by Dunn’s Multiple Comparison Test: *p<0.05; **p<0.01 as depicted by lines.
(TIF)

S7 Fig Effect of the inhibition of HIF-1α inhibitor on lactate production in Mtb-infected M(IL-4) macrophages. Lactate release by M(IL-4) macrophages infected or not with Mtb and treated with PX-478 or its vehicle (DMSO) for 24 h (n = 4). Friedman followed by Dunn’s Multiple Comparison Test: *p<0.05 as depicted by lines.
(TIF)
