# Supplementary figures and images for: Fatty acid oxidation of alternatively activated macrophages prevents foam cell formation, but Mycobacterium tuberculosis counteracts this process via HIF-1α activation
Source: PLoS Pathog. 2020 Oct 1;16(10):e1008929. doi: 10.1371/journal.ppat.1008929 (PMC7553279; doi:10.1371/journal.ppat.1008929)

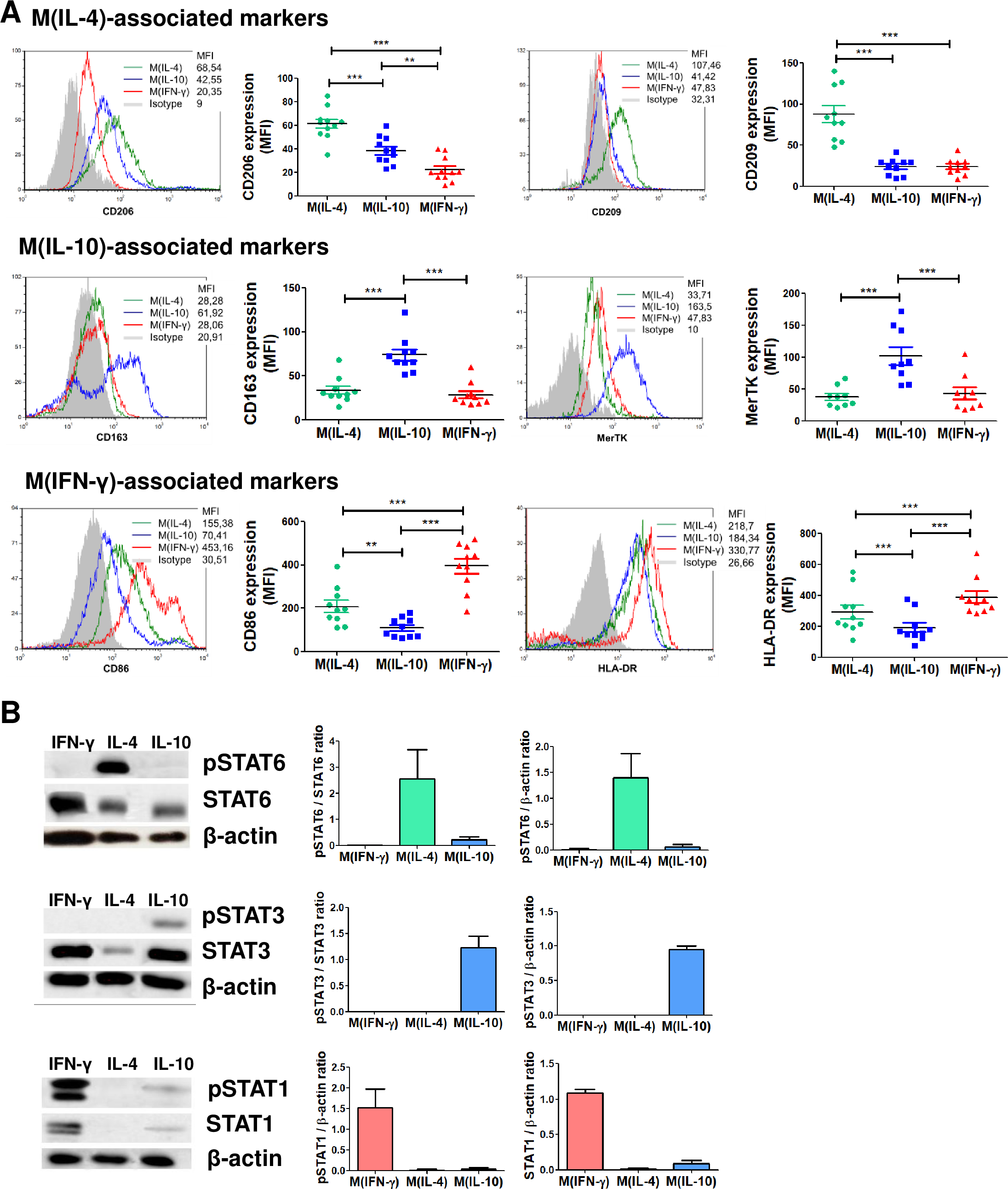

Supplement: S1 Fig — Macrophages were stained with fluorophore-conjugated mAbs PE-anti-CD163 (clone GHI/61), APC-anti-MerTK (clone 590H11G1E3), FITC-anti-CD206 (clone C068C2), PerCP.Cy5.5-anti-CD86 (clone 374216) (all from Biolegend), PE-anti-CD209 (clone, R&D Systems), or FITC-anti-HLA-DR (clone G46-6, BD Biosciences), and in parallel, with the corresponding isotype control antibody. The monocyte-macrophage population was gated according to its Forward Scatter and Size Scatter properties. (A) Median fluorescence intensity (MFI) of CD206, CD209, CD163, MerTK, CD86, and HLA-DR measured by flow cytometry on M(IL-4), M(IL-10) and M(IFN-γ) macrophages. Representative histograms and values of ten independent experiments are shown. Friedman test followed by Dunn’s Multiple Comparison Test: **p<0.01; ***p<0.001; as depicted by lines. (B) Analysis of pSTAT6 (anti-human pY641-STAT6, 1:200 dilution, clone D8S9Y), STAT6 (anti-STAT6, 1:1000 dilution; clone D3H4), pSTAT3 (anti-human pY705-STAT3, 1:1000 dilution, clone D3A7), STAT3 (anti-STAT3, 1:1000 dilution; clone D1A5), pSTAT1 (anti-human pY701-STAT1, 1:1000 dilution, clone D4A7), STAT1 anti-STAT1, 1/500 dilution), all from Cell Signaling Technology, and β-actin protein (1:2000 dilution; ThermoFisher, clone AC-15) expression level by Western Blot and quantification in M(IL-4), M(IL-10) and M(IFN-γ) macrophages. (TIF) [file ppat.1008929.s001.tif]

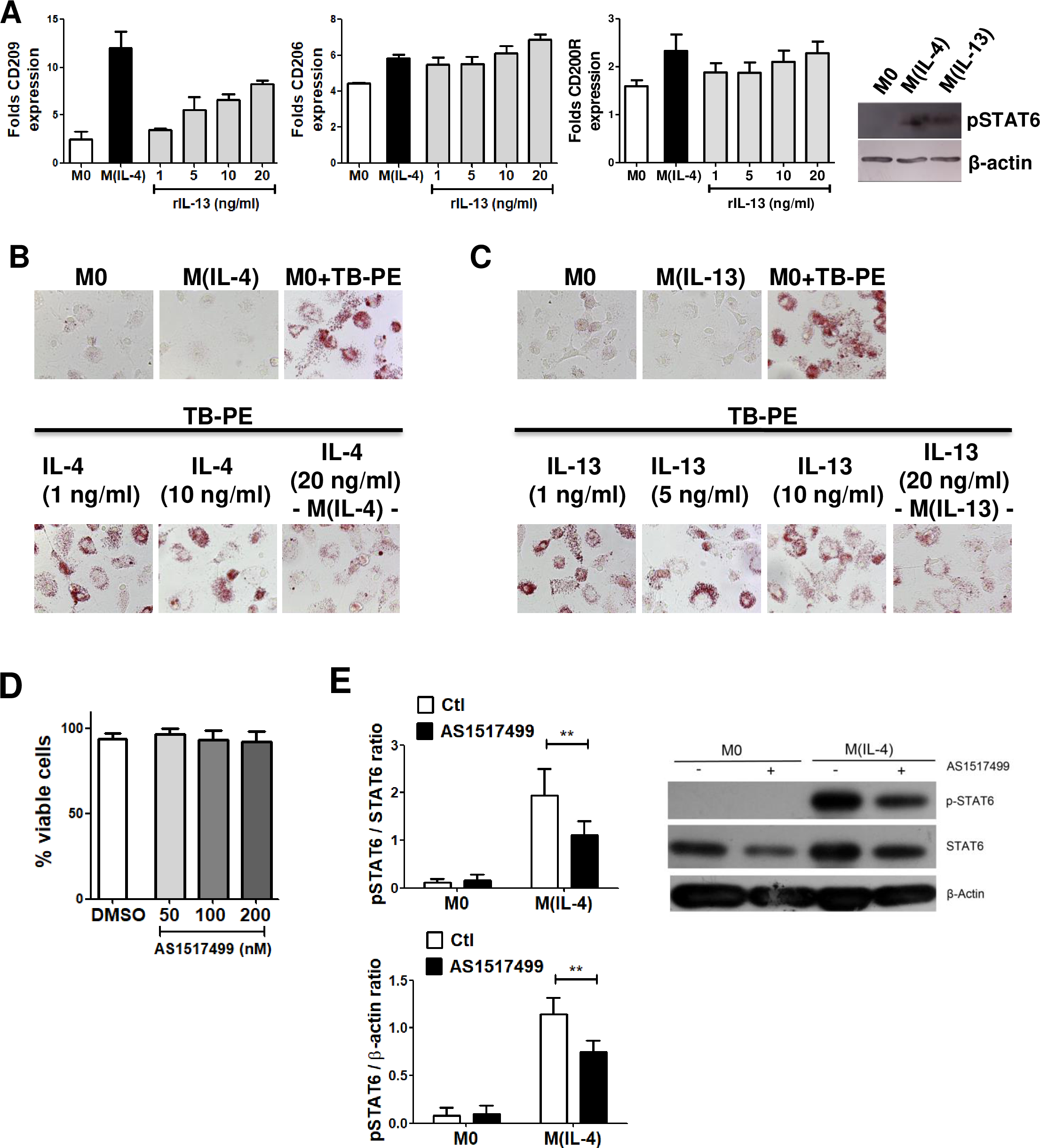

Supplement: S2 Fig — (A) MFI of CD209, CD206, and CD200R measured by flow cytometry on M0, M(IL-4), and macrophages polarized with different amounts of recombinant IL-13. Left panel represents the analysis of pSTAT6 and β-actin protein expression level M0, M(IL-4), and M(IL-13) by Western Blot (B-C) Representative images of ORO staining of macrophages polarized with either different doses of recombinant IL-4 (B) or IL-13 (C) for 48 h and exposed to TB-PE for further 24 h (40x magnification). (D) Cell viability of M(IL-4) macrophages exposed to different amounts of AS1517499 or vehicle. (E) Analysis of pSTAT6, STAT6, and β-actin protein expression level by Western Blot (right panel) and quantifications (left panels, n = 4) in M0 and M(IL-4) macrophages exposed to AS1517499 or vehicle. Wilcoxon signed rank test: *p<0.05 as depicted by lines. (TIF) [file ppat.1008929.s002.tif]

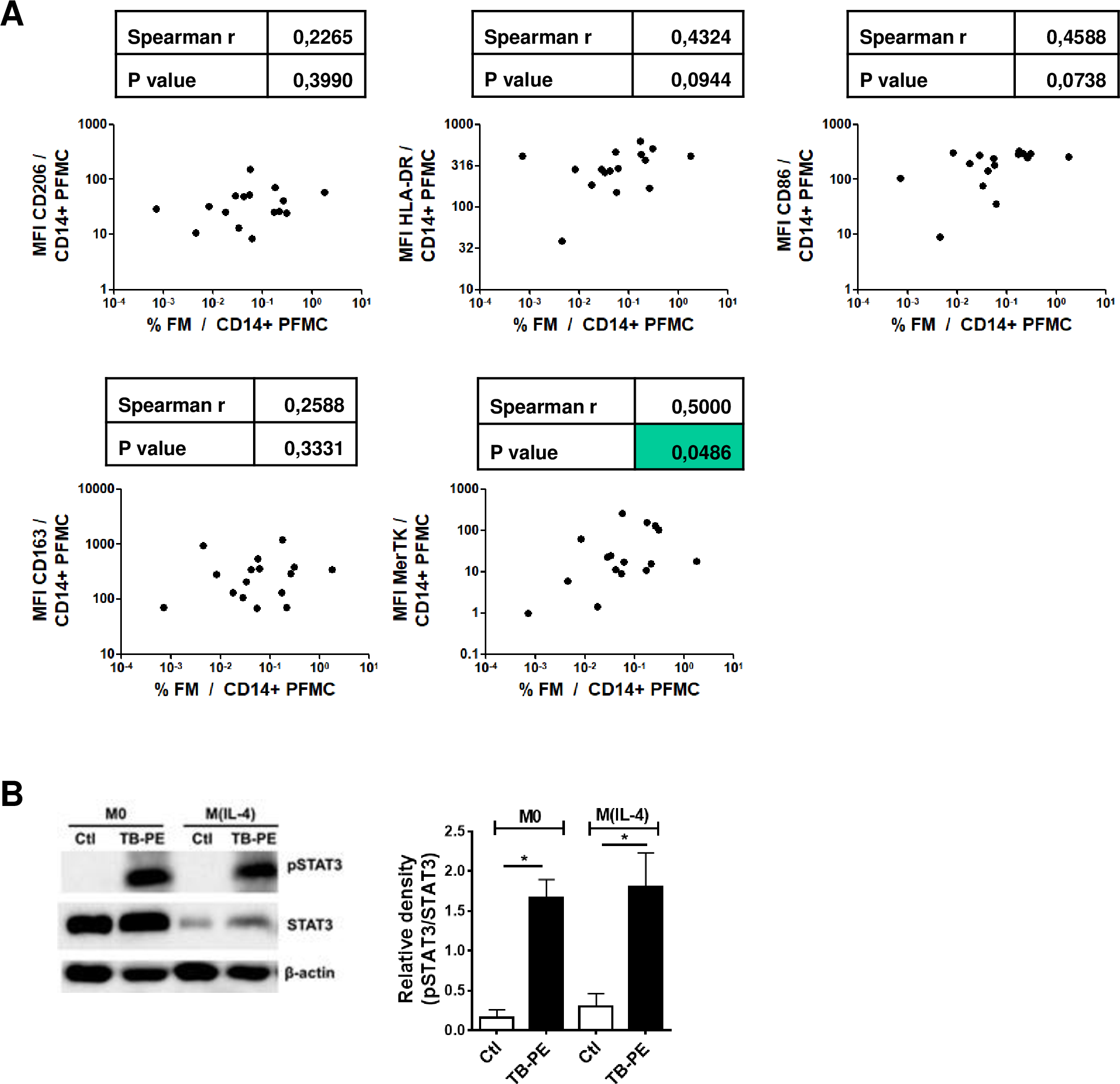

Supplement: S3 Fig — (A) Correlation study between the MFI of CD206, HLA-DR, CD86, CD163, and MerTK cell-surface expression in CD14+ cells from TB pleural cavity and the percentage of lipid-laden CD14+ cells within the pleural fluids mononuclear cells (PFMC) (n = 16) found in individual preparations of TB-PE. Spearman’s rank test. (B) Analysis of pSTAT3, STAT3 and β-actin protein levels by Western Blot (left panel) and quantifications (right panel, n = 4) in M0 and M(IL-4) macrophages treated or not with TB-PE for 24 h (n = 4). Wilcoxon signed rank test: *p<0.05. (TIF) [file ppat.1008929.s003.tif]

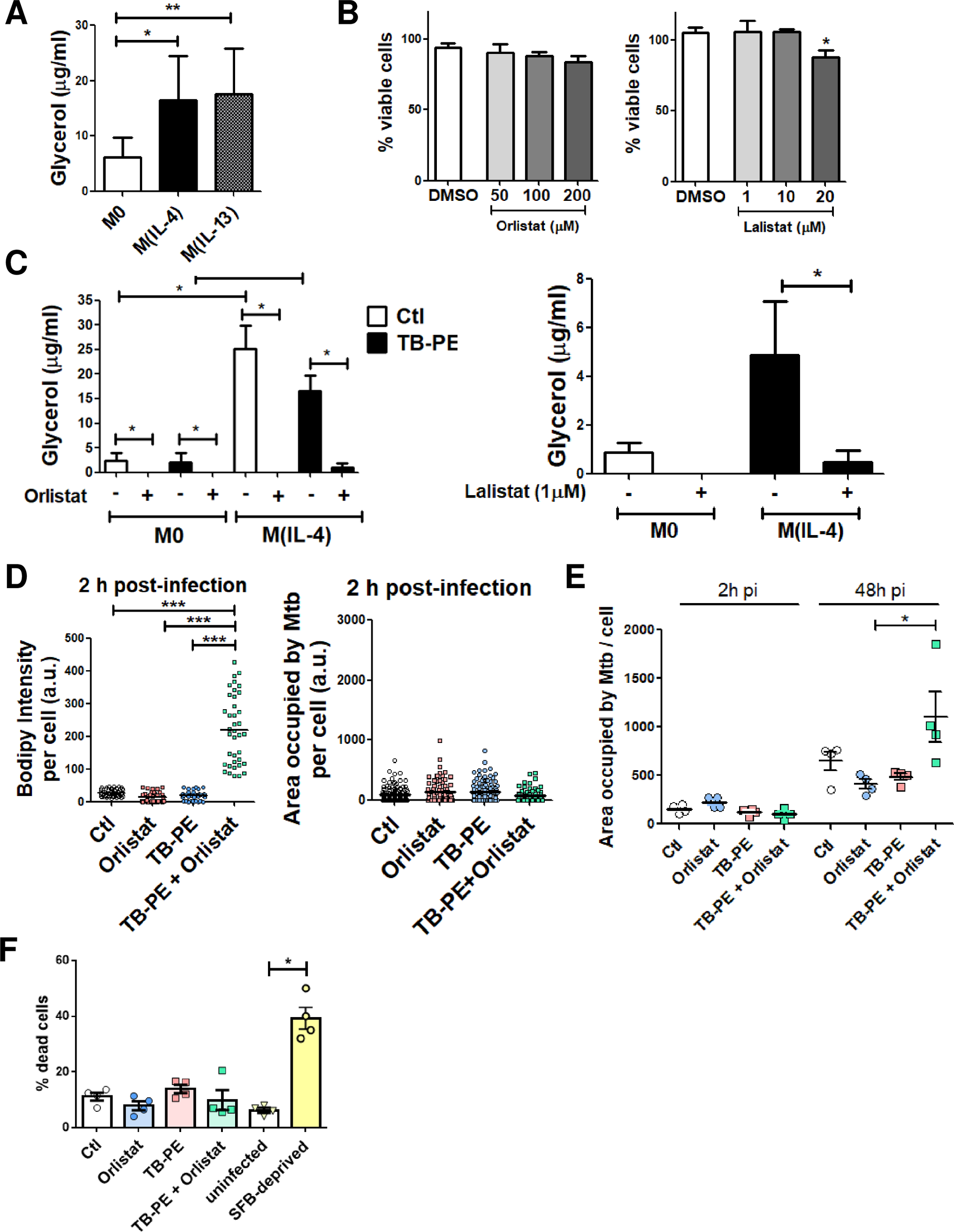

Supplement: S4 Fig — (A) Glycerol release by M0, M(IL-4) and M(IL-13) macrophages (left panel, n = 6). (B) Cell viability of M(IL-4) macrophages exposed to Orlistat (upper panel, n = 4) and Lalistat (lower panel, n = 4). (C) Glycerol release by M0 and M(IL-4) macrophages exposed or not to either Orlistat (left panel, n = 6) or Lalistat (right panel, n = 4). (D-E) M(IL-4) macrophages infected with RFP-Mtb and stained with BODIPY 493/503 at 2 h and 48 h post-infection. (D) BODIPY intensity (left panel) and area occupied by RFP-Mtb (right panel) per cell in z-stacks from confocal laser scanning microscopy images at 2 h post-infection. Each determination represents individual cells of one donor. One way-ANOVA followed by Bonferroni test: ***p<0.001. (E) Area with RFP-Mtb per cell in z-stacks from confocal laser scanning microscopy images. Values are expressed as means of 80–100 cells in four independent experiments. Friedman followed by Dunn’s Multiple Comparison Test: *p<0.05 as depicted by lines. (F) M(IL-4) macrophages were treated or not with TB-PE in the presence or not of Orlistat for 24 h, washed and infected with Mtb (MOI = 5). After 48 h, cells were stained with the fixable vital dye eFluor 780. The percentages of non-viable cells are shown. Mean +/- SEM, N = 4. (TIF) [file ppat.1008929.s004.tif]

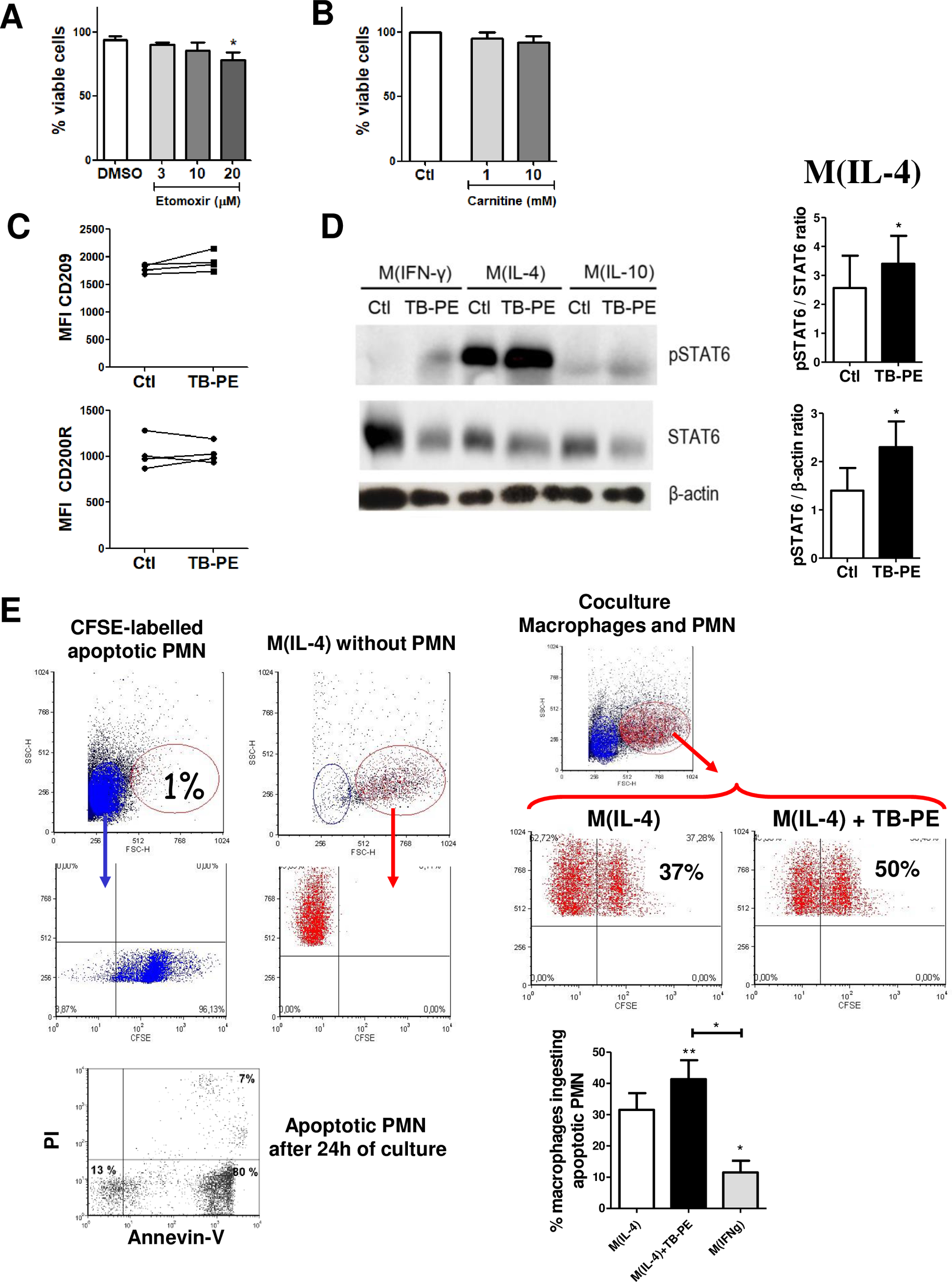

Supplement: S5 Fig — (A) Cell viability of M(IL-4) macrophages exposed to different amounts of Etomoxir (n = 4). (B) Cell viability of M0 macrophages exposed to different amounts of L-carnitine (n = 8). (C) MFI of CD209, and CD200R measured by flow cytometry on M(IL-4) treated or not with TB-PE. (D) Analysis of pSTAT6, STAT6, and β-actin protein expression level by Western Blot (left panel) in M(IFN-γ), M(IL-4), and M(IL-10) macrophages treated or not with TB-PE. Quantifications in M(IL-4) cells (right panels, n = 4). Wilcoxon signed rank test: *p<0.05. (E) CFSE-labelled apoptotic neutrophils (PMN) were cocultured with M(IL-4) macrophages treated or not with TB-PE for 1 h, washed stringently to remove free PMN, and the percentage of macrophages positive for CFSE was assessed. Representative dot blots showing CFSE labeling of apoptotic PMN, M(IL-4), and cocultures are shown. Blue gates represent mainly CFSE labeling of apoptotic PMN while red gates comprise mainly macrophages. Lower panels: Annexin V positive vs propidium iodide negative of apoptotic PMN (left panel) and percentages of M(IL-4) treated or not with TB-PE macrophages ingesting apoptotic PMN (right panel). M(IFN-γ) macrophages were also tested for comparison. (TIF) [file ppat.1008929.s005.tif]

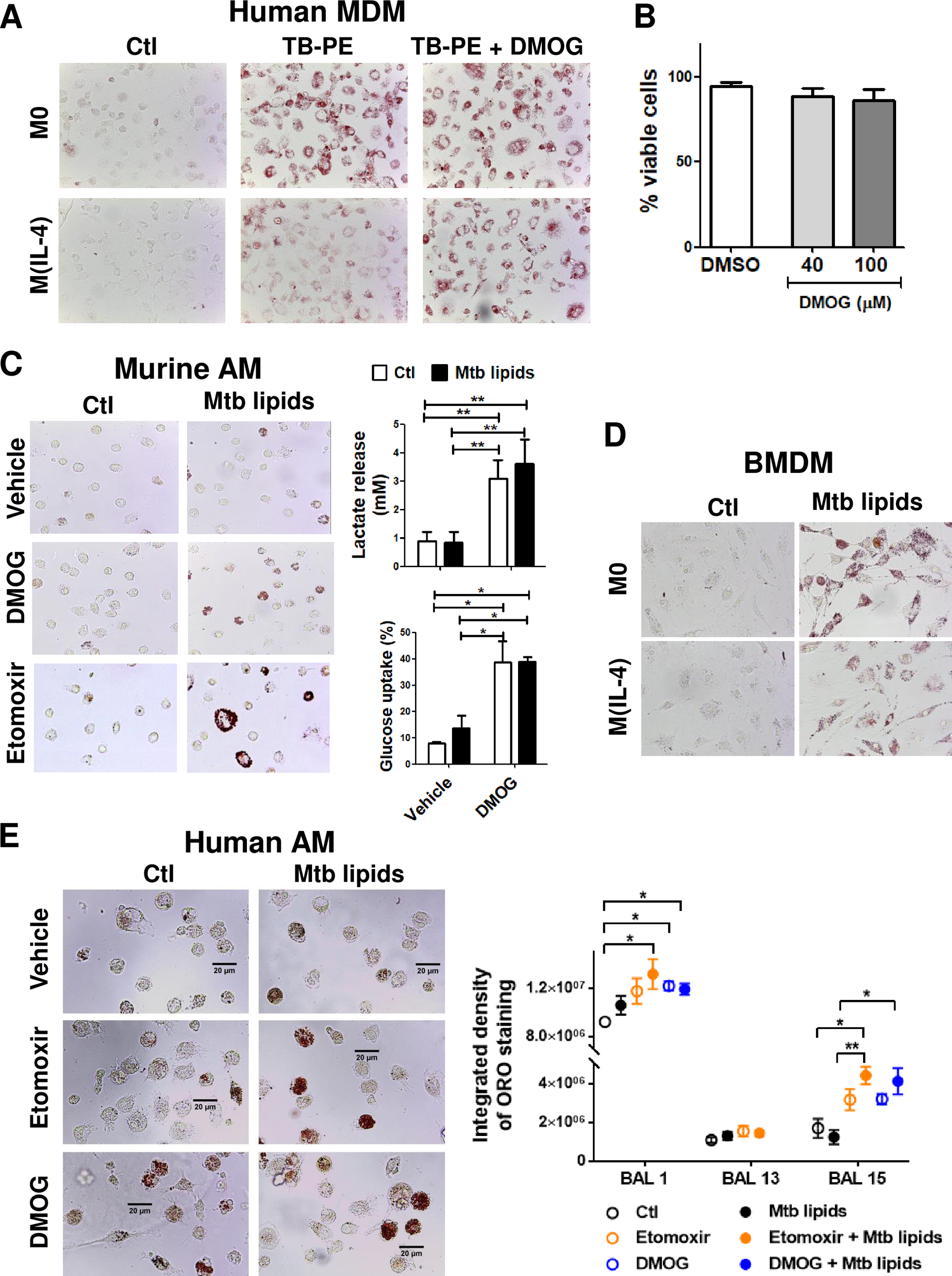

Supplement: S6 Fig — (A) Human macrophages were left untreated (M0) or polarized with IL-4 (M(IL-4)) for 48h, treated or not with the acellular fraction of TB pleural effusions (TB-PE) in the presence or not of DMOG (100 μM) for 24 h and then stained with Oil Red O (ORO). Representative images are shown (40× magnification). (B) Cell viability of M0 macrophages exposed to different amounts of DMOG. (C) Left panel: Representative images (40× magnification) of ORO staining of murine AM treated or not with Mtb lipids in the presence of either DMOG (200 μM) or Etomoxir (3 μM). Right panel: Lactate release and glucose consumption by AM treated or not with Mtb lipids in the presence of DMOG for 24 h (n = 3). One-way ANOVA followed by Bonferroni's Multiple Comparison Test: *p<0.05; **p<0.01, as depicted by lines. (D) Representative images (40× magnification) of ORO staining of murine bone marrow derived macrophages murine (BMDM) unpolarized or polarized towards M(IL-4), exposed or not to Mtb lipids. (E) Leftovers of bronchoalveolar lavages from unidentified patients undergoing bronchoscopy for clinical reasons unrelated to pulmonary infections were collected, according to the protocol approved by the Ethics Committees of the Hospital F.J Muñiz. Bronchoalveolar lavage fluid was centrifuged at 1,400 rpm for 10 minutes. Cells were resuspended at 5 × 10^5^ cells / ml in RPMI 1640 culture media supplemented with FBS, 100 U/ml penicillin, and 100 μg/ml streptomycin. Adherence purification of AM was performed; nonadherent cells were removed by washing after 1 h. Human alveolar macrophages isolated from 3 different donors were treated or not with etomoxir (3 μM) or DMOG (200 nM) for 1 h, prior to Mtb lipids stimulation for further 24 h and then stained with Oil Red O (ORO). Left panel: Representative images (40× magnification), right panel: quantification of ORO staining. Each dot represents the mean of five or six micrograph per condition. Kruskal-Wallis test followed by Dunn’s Multiple Comparison Test: [file ppat.1008929.s006.tif]

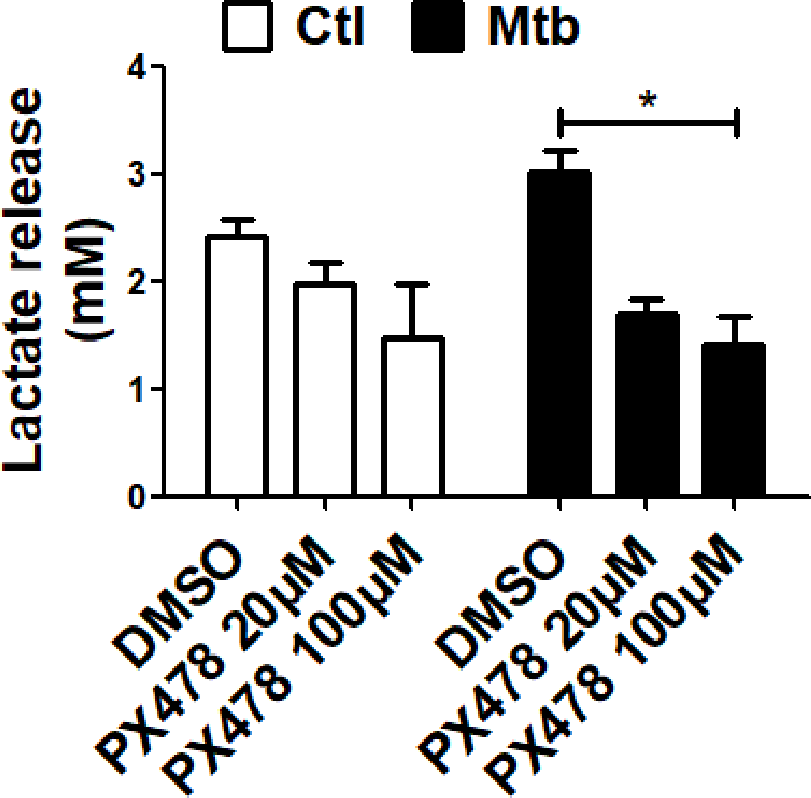

Supplement: S7 Fig — Lactate release by M(IL-4) macrophages infected or not with Mtb and treated with PX-478 or its vehicle (DMSO) for 24 h (n = 4). Friedman followed by Dunn’s Multiple Comparison Test: *p<0.05 as depicted by lines. (TIF) [file ppat.1008929.s007.tif]
